# Distinct functional roles of the GRIP domains in α-, β-, γ-, and δ-ENaC

**DOI:** 10.1016/j.jbc.2026.113336

**Published:** 2026-07-16

**Authors:** Alexandr V. Ilyaskin, Alicia Kißler, Franziska Schreiber, Christoph Korbmacher, Florian Sure

**Affiliations:** Institute of Cellular and Molecular Physiology, Friedrich-Alexander-Universität Erlangen-Nürnberg, Erlangen, Germany

**Keywords:** epithelial sodium channel (ENaC), protein domain, proteolytic channel activation, renal physiology, electrophysiology, site-directed mutagenesis, Xenopus, oocyte

## Abstract

The epithelial sodium channel (ENaC) is a heterotrimer typically composed of three homologous subunits (α, β, and γ). Humans and several other species express an additional δ-subunit that can replace α-ENaC in heterologous expression systems, thereby modifying channel properties. A unique feature of ENaC is its proteolytic activation. Proteases remove autoinhibitory tracts from the Gating Relief of Inhibition by Proteolysis (GRIP) domains of α- and γ-ENaC. Recent ENaC structural data revealed that these tracts occupy specific binding pockets within their respective subunits. Using molecular dynamics simulations, site-directed mutagenesis, and electrophysiological recordings, we identified a cluster of four functionally important aromatic residues within the binding pocket of the γ-autoinhibitory tract in human αβγ-ENaC. These residues were also essential for ENaC inhibition by the synthetic γ-11 peptide, corresponding to the key portion of the γ-inhibitory tract. The aromatic cluster was conserved in α-ENaC, where it mediated the inhibitory effect of the synthetic α-8 peptide. Interestingly, structurally similar GRIP domains were also identified in δ- and β-ENaC. Our data indicate that the GRIP domains of both β- and δ-ENaC lack corresponding autoregulatory tracts. Moreover, we show that proteolytic activation of human δβγ-ENaC results from cleavage of γ-ENaC, but not δ-ENaC, consistent with the absence of autoregulatory activity of the δ-GRIP domain. In summary, this study demonstrates that the GRIP domains play distinct functional roles across different ENaC subunits. Characterization of the inhibitory sites in γ- and α-ENaC may facilitate the development of novel peptidomimetic ENaC inhibitors with potential (patho)physiological implications.

The epithelial sodium channel (ENaC) belongs to the ENaC/degenerin family of non-voltage-gated cation channels (1, 2). The canonical and best-studied variant of the channel is a heterotrimer consisting of an α-, β-, and γ-subunit. αβγ-ENaC is expressed in various epithelial tissues, including the distal nephron and lung epithelia, where it mediates sodium transport across the apical plasma membrane (3). The channel plays a crucial role in maintaining sodium homeostasis, controlling extracellular volume, and regulating long-term blood pressure (4, 5). ENaCs' physiological importance is exemplified by diseases leading to aberrant upregulation of the channel in the kidney, due to hyperaldosteronism (Conn’s syndrome) or gain-of-function ENaC mutations (pseudohyperaldosteronism, Liddle’s syndrome), both causing severe hypertension, hypokalemia, and metabolic alkalosis (6, 7, 8). Conversely, insufficient renal ENaC function resulting from hypoaldosteronism (Addison’s disease) or loss-of-function ENaC mutations (pseudohypoaldosteronism type I, PHA1) leads to hypotension, hyperkalemia, and acidosis (9, 10, 11). Beyond its well-established role in these severe disorders, abnormally elevated ENaC function in the kidney is thought to contribute to the pathophysiology of essential salt-sensitive hypertension (12). Furthermore, increased ENaC activity is discussed as a factor in nephrotic syndrome that contributes to sodium retention and edema formation (13, 14, 15, 16). Besides its pivotal role in the kidney, ENaC also controls the surface liquid volume in both the airway and alveolar epithelia (17). Here, impaired ENaC function can lead to respiratory distress and pulmonary edema (18, 19, 20), whereas exaggerated ENaC activity likely contributes to the pathophysiology of cystic fibrosis (21, 22, 23, 24, 25, 26). In summary, the multitude of disorders associated with αβγ-ENaC underscores its important physiological role and clinical relevance.

In many species, including humans, an additional δ-subunit has been identified (27, 28, 29, 30, 31). It can replace the α-subunit to form functional δβγ-channels in heterologous expression systems with altered biophysical properties compared to the αβγ-configuration of the channel (28, 32). Notably, no recordings of functional δβγ-ENaC from native tissues have been published, and the (patho)physiological role of the δ-subunit remains largely unclear. An interesting hypothesis regarding the possible role of δβγ-ENaC in salt sensing has recently been proposed (31). According to the authors, δβγ-ENaC expressed in taste buds may contribute to the detection of high sodium concentrations due to its unique biophysical properties, particularly due to a reduced Na^+^ self-inhibition (30, 31). The latter is an intrinsic feedback mechanism by which elevated extracellular Na^+^ reduces channel open probability. In line with this hypothesis is the observation that the *Scnn1d* gene, which encodes the δ-subunit, emerged in tetrapod ancestors during the water-to-land transition and became non-functional in many marine mammal species, which are constantly exposed to a high-salt marine environment (31).

Recently, cryo-electron microscopy (cryo-EM) structures of ENaC in both the αβγ- (33, 34) and δβγ-configuration (35) have been resolved. These structural data indicate that three homologous ENaC subunits can assemble into heterotrimeric ion channels (αβγ-ENaC or δβγ-ENaC) that share substantial structural similarity with the related acid-sensing ion channels (ASICs) (2). Each ENaC subunit is characterized by a large extracellular loop that resembles a hand holding a ball with palm, thumb, knuckle, finger, and β-ball domains. While these domains are also found in the related ASICs, a distinctive feature of ENaC subunits are the so-called Gating Relief of Inhibition by Proteolysis (GRIP) domains that are involved in a unique mechanism of channel regulation by proteases (33, 34). Indeed, proteases activate human ENaC in both the αβγ- (36, 37, 38, 39, 40, 41) and the δβγ-configuration (28, 30). It is well established that proteolytic cleavage results in the removal of autoinhibitory tracts 26 and 43 residues in length from the α-GRIP and γ-GRIP domains, respectively (38, 42, 43). Importantly, previous studies demonstrated that a proximal portion of the autoinhibitory tracts, hereafter referred to as the P1 segment, contains the key residues that mediate their inhibitory effect (44, 45). In line with this, application of small synthetic inhibitory peptides corresponding to the P1 sequence of α-ENaC (α-8; LPHPLQRL) or γ-ENaC (γ-11; RFSHRIPLLIF) has been shown to inhibit ENaC-mediated currents (44, 45, 46). Despite detailed structural characterization, the functional importance of individual residues forming the binding sites for the P1 segments remains to be clarified. In contrast to α- and γ-ENaC subunits, β-ENaC is not subject to proteolytic processing. Furthermore, incorporation of δ- instead of α-ENaC into the ENaC heterotrimer has been shown to affect proteolytic channel activation in a species-specific manner. However, the underlying molecular mechanisms remain largely unclear (28, 30, 32). Notably, a GRIP domain with a similar structural organization, resembling a β-sheet blanket covering the α2-helix of the finger domain, is present in both the β- and δ-subunits. However, the functional relevance of the GRIP domains in β- and δ-ENaC has yet to be determined.

The aim of the present study was to provide new insights into the molecular mechanisms underlying proteolytic ENaC activation in its αβγ- and δβγ-configuration. We focused on human ENaC throughout the study because its structure has been resolved by cryo-EM (33, 34, 35). This allowed us to perform structure-based molecular dynamics (MD) simulations, which we combined with functional experiments to identify critical amino acid residues within the binding sites for the P1 segments in α- and γ-ENaC. Furthermore, we demonstrated that, in contrast to α- and γ-ENaC, the GRIP domains of β- and δ-ENaC do not contain autoregulatory tracts. Finally, we revealed that cleavage of the δ-subunit does not contribute to proteolytic activation of δβγ-ENaC, which depends solely on γ-ENaC cleavage. In conclusion, our findings highlight distinct functional roles of the GRIP domains in different ENaC subunits.

## Results

### Structural similarities of the GRIP-domains in α-, β-, γ-, and δ-ENaC

**Figure 1:**
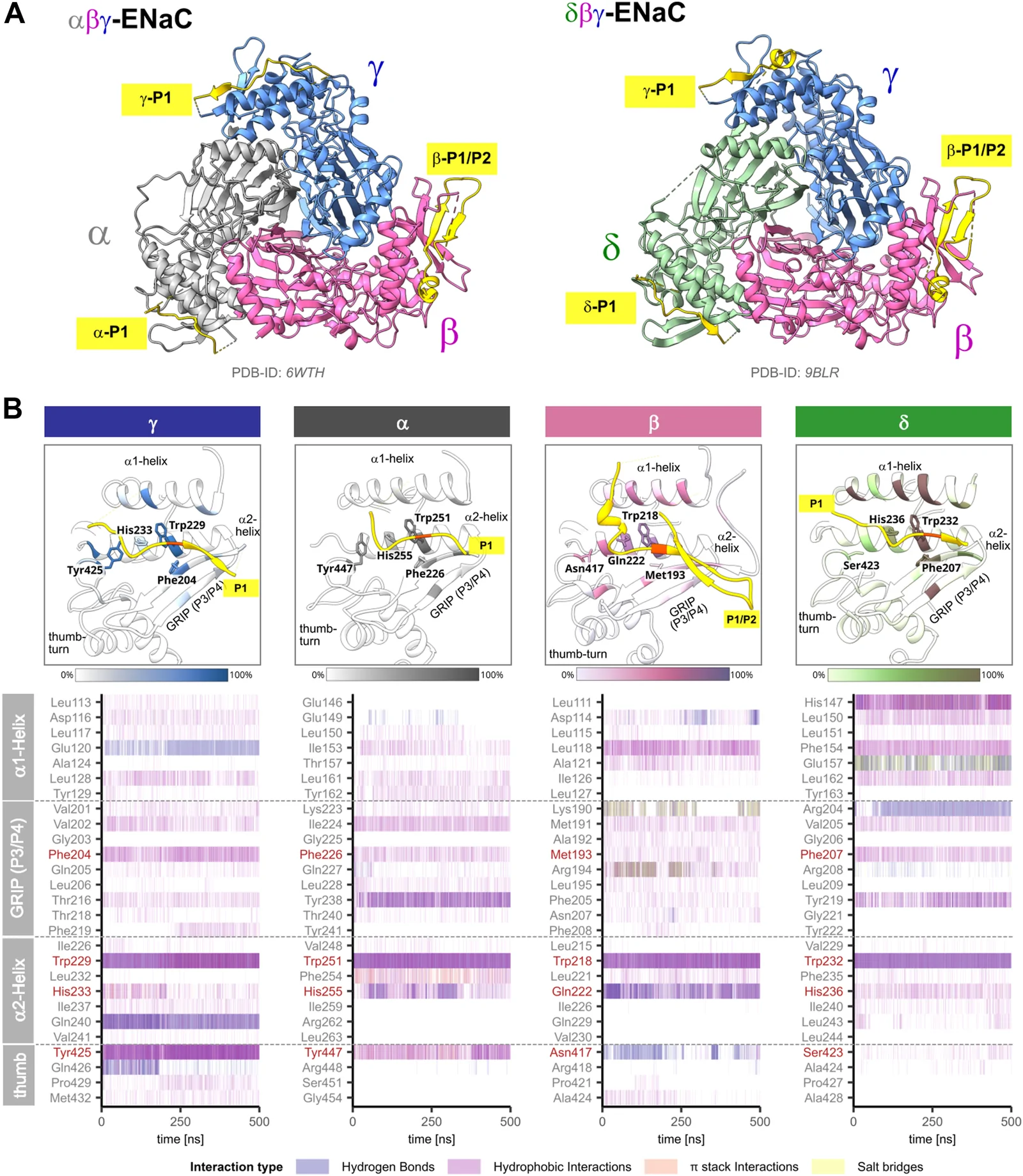
**Molecular dynamics (MD) simulations of αβγ-ENaC and δβγ-ENaC predict key interacting residues for the P1 segments of the GRIP domains within their respective binding pockets.***A*, top view of human ENaC in its αβγ (*left panel*) and δβγ (*right panel*) configuration in ribbon representation, generated using atomic coordinates from PDB entries 6WTH (34) and 9BLR (35), respectively. Individual ENaC subunits are displayed in different colors as indicated. The P1 segment of the GRIP domain in α-, γ-, and δ-ENaC, as well as the P1/P2 segment of the GRIP domain in β-ENaC, are colored in yellow. Dotted lines indicate unresolved protein regions. *B*, *upper panels* show the binding sites for the P1 segments in the γ-, α-, and δ-subunit and that for the P1/P2 segment in the β-subunit on an expanded scale. The binding sites are formed by residues of the finger (α1- and α2-helix), GRIP (P3/P4), and thumb domains as indicated. Individual residues are colored according to the scheme shown below the structure, representing the stability of their interactions with the P1 segment during 500-ns MD simulations (0–100% of the simulation time, where 100% indicates persistent interactions throughout the simulation and 0% indicates no interactions; colormaps from (80)). *Lower panels* depict interactions formed between each P1 binding-site residue (y-axis) and the P1 segment during the MD simulations. For γ-, α-, and β-ENaC, MD was conducted using the 6WTH structure (αβγ-ENaC configuration). For δ-ENaC, MD was conducted using the 9BLR structure (δβγ-ENaC configuration). Interaction types are indicated by different colors as specified below. Each line corresponds to the formation of at least one interaction of the specific type per trajectory frame, and darker color intensity indicates multiple interactions of the same type per trajectory frame. In γ- and α-ENaC, four conserved aromatic residues forming the P1 binding site are highlighted in red in the *lower panels,* and shown in stick representation in the *upper panels*. For comparison, the corresponding residues in β- and δ-ENaC are presented in the same manner. Conserved leucine residues in the middle of the P1 segments, which form stable interactions with the conserved tryptophan residues of the P1 binding site, are colored in *orange*.

Available cryo-EM structures of ENaC in both αβγ- and δβγ-configurations resolved putative P1 segments of the GRIP domains not only in α- and γ-ENaC but also in β- and δ-ENaC. An additional β-sheet, referred to as a P2 segment in the original cryo-EM work (33), was only resolved in the β-subunit (Fig. 1*A*). Notably, in all four subunits the regions connecting the P1 segments (and the P1/P2 segment in β-ENaC) to the rest of the protein remained unresolved, likely due to their flexible and unstructured nature. This indicates that noncovalent interactions are critical for stabilizing the P1 segments (P1/P2 in β-ENaC) in their respective positions. Indeed, according to the structural data these segments occupy shallow binding pockets on the molecular surface of ENaC, formed by the α1- and α2-helices of the finger domain, the P3-P4 regions of the GRIP domain, and a short loop at the top of the thumb domain (Fig. 1, *A* and *B*). To assess whether the noncovalent interactions between the P1 strands and their respective binding pockets are sufficient to prevent dissociation of the P1 (P1/P2 in β-ENaC) segments from the ENaC core, we performed two replica 500-ns atomistic molecular dynamics (MD) simulations of αβγ-ENaC and δβγ-ENaC, using initial atomic coordinates from Protein Data Bank (PDB) structures 6WTH (34) and 9BLR (35), respectively. Our goal was to identify key interactions between the P1 segments and their respective binding pockets across all four ENaC subunits.

Surprisingly, the MD simulations revealed that each P1 segment remained stably associated with its binding pocket throughout the entire simulation (Fig. S1). The binding was stabilized by multiple residues forming predominantly hydrophobic interactions and hydrogen bonds (Figs. 1*B* and S2). Notably, in both the γ- and α-subunits, the P1 segments were coordinated by four conserved aromatic residues located in the GRIP domain (γPhe204 in γ-ENaC; αPhe226 in α-ENaC), the finger α2-helix (γTrp229 and γHis233 in γ-ENaC; αTrp251 and αHis255 in α-ENaC), and the thumb domain (γTyr425 in γ-ENaC; αTyr447 in α-ENaC). A similar interaction pattern was observed in the δ-subunit, where three conserved aromatic residues (δPhe207, δTrp232, and δHis236) contributed significantly to P1 coordination. However, in contrast to the γ- and α-subunits, the contribution of the thumb domain in δ-ENaC was markedly reduced, while that of the α1-helix in the finger domain became more prominent. In β-ENaC, only a single aromatic residue, βTrp218, located at the center of the α2-helix, participated in P1 stabilization, whereas other aromatic positions were functionally replaced by polar (βAsn417, βGln222) and hydrophobic (βMet193) residues. Notably, across all four ENaC subunits, the conserved tryptophan residues within the finger α2-helix (γTrp229; αTrp251; βTrp218; δTrp232) formed stable hydrophobic contacts with conserved leucine residues of the corresponding P1 segments (γLeu160; αLeu188; βLeu150; δLeu193), suggesting functional importance of these interactions (Fig. 1*B*).

To conclude, our MD simulations revealed that the P1 segments formed stable interactions with their respective binding pockets across all ENaC subunits. In γ- and α-ENaC, four conserved aromatic residues appear to play an important role in P1 coordination. Furthermore, the overall structural similarity of the GRIP domains and the presence of conserved Trp-Leu interactions in all four subunits suggest that the P1 segments may be functionally important not only in the γ- and α- but also in the δ- and β-subunits.

### Conserved aromatic residues within the binding site of the P1 segment in γ-ENaC are critical for channel inhibition by the γ-autoinhibitory tract and the synthetic γ-11 peptide

Our MD simulations predicted that four conserved aromatic residues (γPhe204, γTrp229, γHis233, and γTyr425) in γ-ENaC contribute to P1 coordination. Since the P1 segment is part of the γ-autoinhibitory tract and contains key inhibitory residues, the conserved aromatic residues forming the P1 binding site likely play a crucial role in proteolytic ENaC activation and inhibition by the synthetic γ-11 peptide (RFSHRIPLLIF).

To validate these predictions, wild-type (WT) human αβγ-ENaC and mutant channels, in which the predicted critical residues were individually substituted with alanine, were heterologously expressed in *Xenopus laevis* oocytes. ENaC-mediated currents were measured using the two-electrode voltage clamp (TEVC) technique. For each experimental group, we assessed baseline currents, the stimulatory effect of the prototypical protease chymotrypsin, and the inhibitory effect of the synthetic γ-11 peptide at two concentrations (1 and 10 μM).

**Figure 2:**
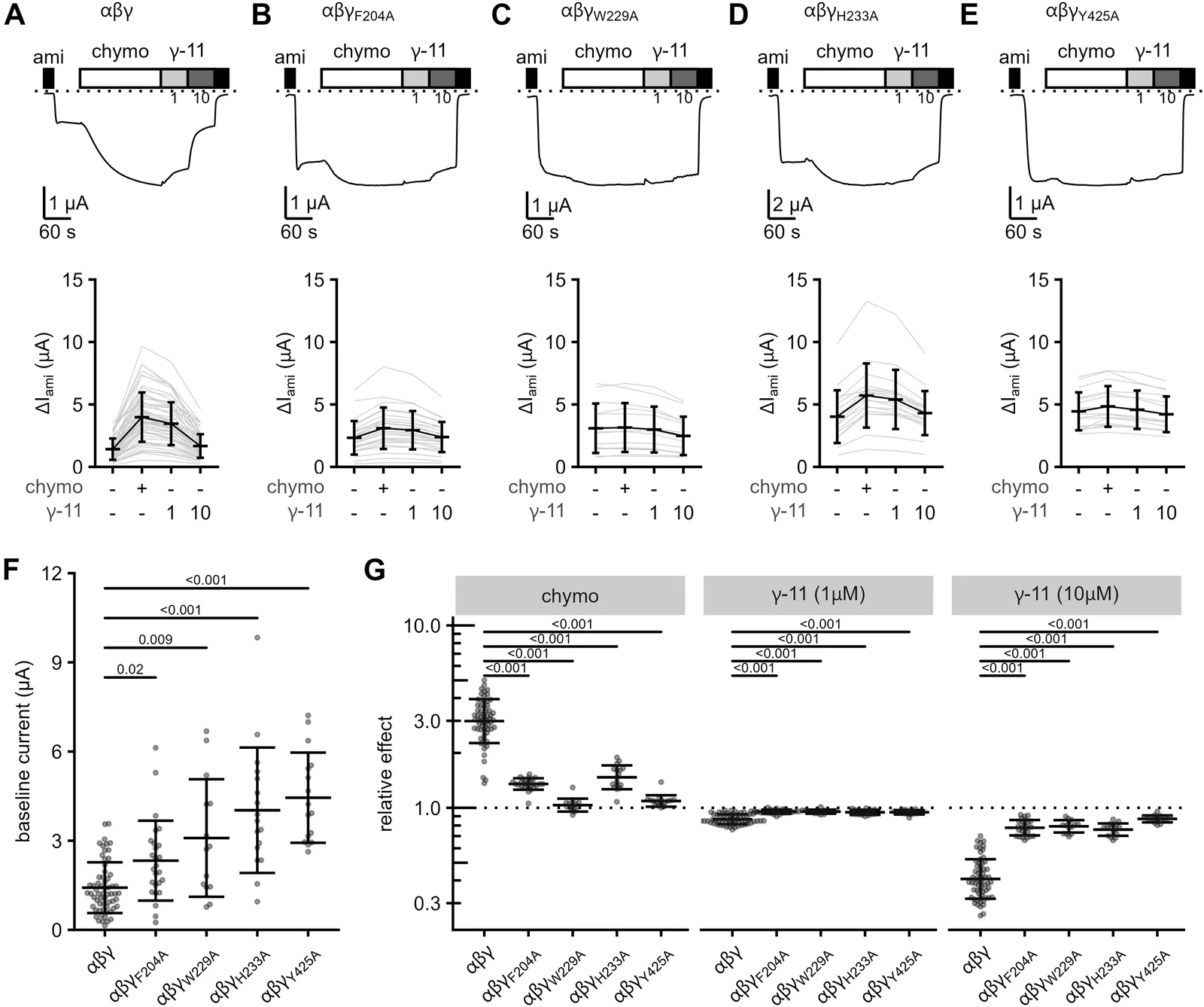
**Substitution of aromatic residues within the P1 binding site of γ-ENaC (γF204, γW229, γH233, γY425) with alanine mimics proteolytic activation of the channel and reduces inhibition by the γ-11 peptide.***A-E,upper panels,* A representative whole-cell current trace is shown for an oocyte co-expressing human wild-type α- and β-ENaC together with the wild-type γ-subunit (αβγ; *A*) or the corresponding γ-ENaC mutant (αβγ_F204A_, αβγ_W229A_, αβγ_H233A_, or αβγ_Y425A_; *B*-*E*). Amiloride (ami, 2 μM), chymotrypsin (chymo, 2 μg/ml) and the γ-11 peptide (at the indicated concentration in μM) were present in the bath solution as indicated by *black*, *white* and *gray shaded* bars, respectively. Dotted lines correspond to the zero current level. *Lower panels* ENaC-mediated amiloride-sensitive whole-cell current values (ΔI_ami_) were determined from similar experiments as shown in the *upper panels* by subtracting the current level reached at the end of each solution application step from the current level in the presence of amiloride. *Gray* lines connect data points obtained in an individual oocyte. Mean ± SD are shown in *black* (*A*: N = 9, n = 62; *B*: N = 4, n = 26; *C*: N = 3, n = 14; *D*: N = 3, n = 17; *E*: N = 2, n = 17; N indicates the number of different batches of oocytes, n indicates the number of individual oocytes studied per experimental group). *F*, Baseline ΔI_ami_ values before application of chymotrypsin were obtained for different ENaC constructs in the same experiments as shown in *A*-*E*. *p* values were calculated using Kruskal-Wallis with Dunn’s *post hoc* test. *G*, Relative effects of chymotrypsin or the γ-11 peptide at the indicated concentrations were calculated for different ENaC constructs using ΔI_ami_ values from the same experiments as shown in *A*-*E* and are displayed on a logarithmic scale. Dotted line indicates relative effect of one (no effect). The values above and below this line correspond to stimulation and inhibition, respectively. *p* values were calculated using Kruskal-Wallis with Dunn’s *post hoc* test of log-transformed values.

In an oocyte expressing WT ENaC, washout of amiloride (2 μM), which blocks ∼90% of the channel activity at this concentration (47), resulted in detectable sodium inward currents (Fig. 2*A*). Subsequent application of chymotrypsin further stimulated these currents by ∼3-fold (Fig. 2, *A* and *G*). Under these conditions, chymotrypsin cleaves the autoinhibitory tract containing the P1 segment from the γ-subunit, increasing the channel’s open probability (P_o_) close to one (28, 39, 48, 49). Following chymotrypsin treatment, sequential application of the synthetic γ-11 inhibitory peptide in two concentrations (1 and 10 μM) reduced ENaC currents to the level measured before proteolytic channel activation. Finally, application of amiloride almost completely blocked channel activity (Fig. 2*A*).

Each of the ENaC mutants exhibited significantly increased baseline currents (Fig. 2, *B*–*F*). Moreover, the mutations either reduced (γF204A, γH233A) or nearly abolished (γW229A, γY425A) the stimulatory effect of chymotrypsin (Fig. 2, *B*–*E*, *G*). Western blot analysis of cell surface and intracellular γ-ENaC expression revealed no substantial differences between WT and mutant channels (Fig. S3), indicating that the increased currents were not due to altered γ-ENaC protein expression. Likewise, the cleavage pattern of γ-ENaC before and after chymotrypsin treatment was not significantly affected by the alanine substitutions, suggesting that these substitutions neither enhanced endogenous proteolytic processing of the γ-subunit, nor impaired chymotrypsin-mediated γ-ENaC cleavage. Collectively, these results indicate that the alanine substitutions increased the channel’s P_o_ by disrupting critical interactions between the γ-autoinhibitory tract and its binding site within γ-ENaC. In line with this, the inhibitory effect of the γ-11 peptide applied after chymotrypsin was also markedly reduced in all ENaC mutants (Fig. 2, *B*–*E*, *G*), indicating that the synthetic peptide acts *via* the same binding site as the γ-autoinhibitory tract.

**Figure 3:**
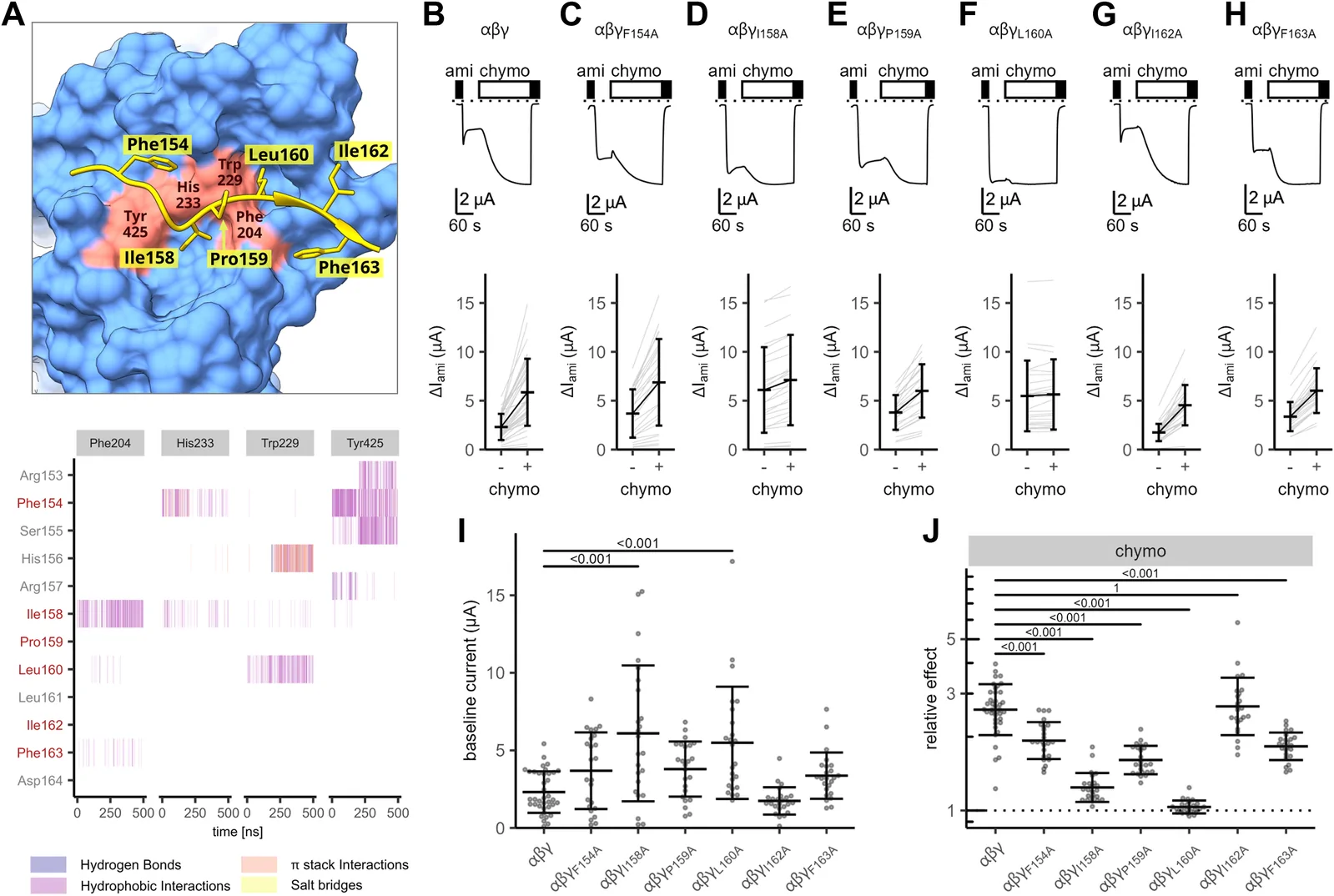
**Residues γI158 and γL160 withing the P1 segment of γ-ENaC are critically involved in mediating its inhibitory effect.***A*, *upper panel,* The P1 segment in *yellow* and ribbon representation bound to the γ-subunit in surface representation is shown. Four functionally important aromatic residues within the P1 binding site are highlighted in *salmon*. Side chains of the P1 residues that were functionally assessed in *C–J* are depicted in stick representation. *Lower panel* Interactions formed between the side chains of the P1 segment residues (y-axis) and the four aromatic P1 binding site residues (Phe204, His233, Trp229, and Tyr425) during the 500-ns MD simulations are depicted as described in Figure 1*B*. Residues of the P1 segment (y-axis) that were functionally assessed in *C*-*J* are highlighted in *red*. *B-H*, *Upper panels*, A representative whole-cell current trace is shown for an oocyte co-expressing human wild-type α- and β-ENaC together with the wild-type γ-subunit (αβγ; *B*) or the corresponding γ-ENaC mutant (αβγ_F154A_, αβγ_I158A_, αβγ_P159A_, αβγ_L160A_, αβγ_I162A_, or αβγ_F163A_; *C-H*). Amiloride (ami, 2 μM) and chymotrypsin (chymo, 2 μg/ml) were present in the bath solution as indicated by *black* and *white* bars, respectively. Dotted lines correspond to the zero current level. *Lower panels*, ΔI_ami_ values were determined before (−) and after (+) chymotrypsin application from similar experiments as shown in the *upper panels* as described in Figure 2. *Gray* lines connect data points obtained in an individual oocyte. Mean ± SD are shown in black (*B*: N = 5, n = 38; *C–H*: N = 3, n = 24; N indicates the number of different batches of oocytes, n indicates the number of individual oocytes studied per experimental group). *I*, baseline ΔI_ami_ values before application of chymotrypsin were obtained for different ENaC constructs in the same experiments as shown in *B*-*H*. *p* values were calculated using Kruskal-Wallis with Dunn’s *post hoc* test. *J*, Relative stimulatory effect of chymotrypsin was calculated for different ENaC constructs using ΔI_ami_ values from the same experiments as shown in *B*-*H* and are displayed on a logarithmic scale. Dotted line indicates relative effect of one (no effect). *p* values were calculated using one-way ANOVA with Bonferroni *post hoc* test of log-transformed values.

Closer inspection of the P1 binding site suggested that the cluster of aromatic residues (γPhe204, γTrp229, γHis233, and γTyr425) contributes to the formation of two small pockets on the molecular surface, which are occupied by the hydrophobic side chains of γIle158 and γLeu160, located approximately in the middle of the P1 segment (Fig. 3*A*, *upper panel*). These hydrophobic side chains may therefore act like a plug, anchoring the P1 segment to its binding site. During the MD simulations, γIle158 formed hydrophobic interactions with all four aromatic residues, with γPhe204 appearing to play the dominant role. γLeu160 interacted mainly with γTrp229 (Fig. 3*A*, *lower panel*). Although the side chain of γPro159, located between γIle158 and γLeu160, did not directly participate in interactions with the binding site (Fig. 3*A*, *lower panel*), it likely stabilized the local conformation of the γIle158 – γLeu160 peptide stretch due to its unique cyclic structure, which restricts backbone flexibility. The aromatic cluster also contributed to the coordination of both the proximal (γArg153 – γSer155) and distal (γIle162 – γAsp164) ends of the P1 segment, with γTyr425 and γPhe204 residues interacting with the proximal and distal regions, respectively (Fig. 3*A*, *lower panel*).

In the light of these predictions, we tested the hypothesis that alanine substitutions of γIle158 or γLeu160 disrupt interactions between the γ-autoinhibitory tract and its binding site. Baseline currents produced by these mutant channels were significantly increased by approximately 2.5-fold (Fig. 3, *D*, *F* and *I*). Moreover, chymotrypsin-mediated ENaC stimulation was strongly reduced for γI158A (Fig. 3, *D* and *J*) and nearly abolished for γL160A (Fig. 3, *F* and *J*), indicating that these alanine substitutions mimicked proteolytic channel activation. Notably, these mutations neither affected proteolytic processing of the γ-subunit by chymotrypsin nor substantially altered γ-ENaC protein expression (Fig. S4), indicating that their effects resulted from increased P_o_. Alanine substitutions of γPro159, γPhe154, or γPhe163 also produced similar effects, although they were less pronounced (Fig. 3, *C* and *E*, *H*–*J*). In contrast, the γI162A mutant exhibited baseline currents and chymotrypsin-mediated activation (Fig. 3, *G*, *I* and *J*) similar to WT ENaC (Fig. 3, *B*, *I* and *J*). This finding is consistent with the structural data showing that the γI162 side chain points away from the P1 binding site and does not interact with the aromatic cluster (Fig. 3*A*).

Collectively, functional analyses of residues belonging to the P1 binding site (Fig. 2) and to the P1 segment (Fig. 3) provided consistent results that suggest a critical role for the aromatic cluster (γPhe204, γTrp229, γHis233, and γTyr425) in mediating channel inhibition by both the γ-autoinhibitory tract and the synthetic γ-11 peptide.

### Functional relevance of conserved aromatic residues within the binding site of the P1 segment in α-ENaC

Our MD simulations (Figs. 1*B* and S2) revealed that, similar to the γ-subunit, four aromatic residues conserved in the α-subunit (αPhe226, αTrp251, αHis255, and αTyr447) stabilize the P1 tract within its binding site, suggesting their functional importance in mediating the inhibitory effects of the α-autoinhibitory tract and the synthetic α-8 peptide (LPHPLQRL) on ENaC. At the oocyte cell surface, however, WT α-ENaC is expressed in a fully proteolytically cleaved form due to intracellular furin-mediated cleavage and thus lacks the autoinhibitory tract (36, 37). Therefore, we focused our investigation on whether these aromatic residues contribute to the channel inhibition by the synthetic α-8 peptide.

**Figure 4:**
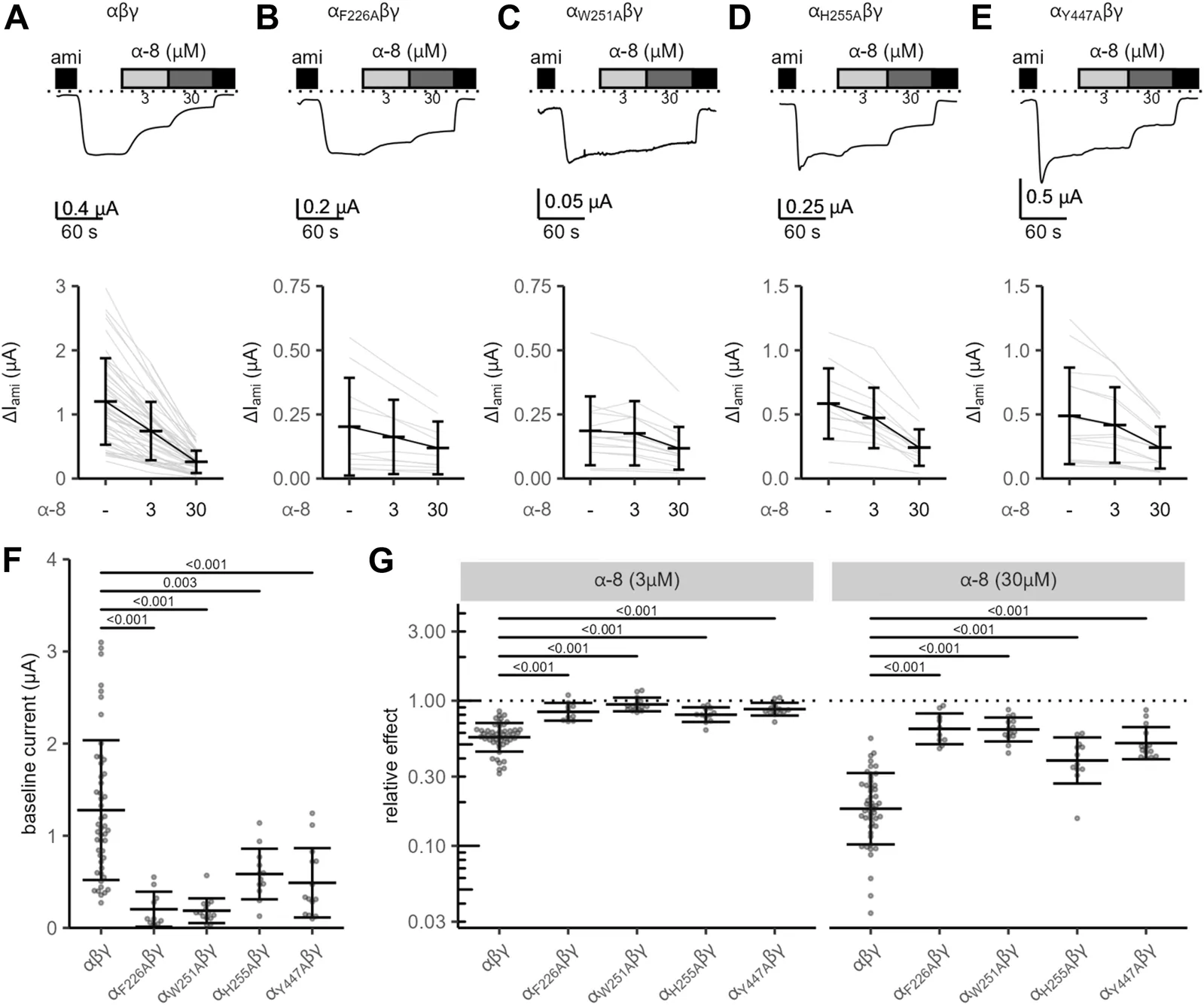
**Aromatic residues within the P1 binding site of α-ENaC (αF226, αW251, αH255, αY447) are critical for channel inhibition by the α-8 peptide.***A-E*, *upper panels,* A representative whole-cell current trace is shown for an oocyte co-expressing human wild-type β- and γ-ENaC together with the wild-type α-subunit (αβγ; *A*) or the corresponding α-ENaC mutant (α_F226A_βγ, α_W251A_βγ, α_H255A_βγ, or α_Y447A_βγ; *B*-*E*). Amiloride (ami, 2 μM) and the α-8 peptide (at the indicated concentration in μM) were present in the bath solution as indicated by *black* and *gray* shaded bars, respectively. Dotted lines correspond to the zero current level. *Lower panels* ΔI_ami_ values were determined from similar experiments as shown in the *upper panels* as described in Figure 2. *Gray* lines connect data points obtained in an individual oocyte. Mean ± SD are shown in *black* (*A*: N = 7, n = 49; *B*: N = 2, n = 10; *C*: N = 2, n = 14; *D*: N = 2, n = 12; *E*: N = 2, n = 14; N indicates the number of different batches of oocytes, n indicates the number of individual oocytes studied per experimental group). *F*, Baseline ΔI_ami_ values before application of the α-8 peptide were obtained for different ENaC constructs in the same experiments as shown in *A*-*E*. *p* values were calculated using Kruskal-Wallis with Dunn’s *post hoc* test. *G,* Relative inhibitory effects of the α-8 peptide at the indicated concentration were calculated for different ENaC constructs using ΔI_ami_ values from the same experiments as shown in *A*-*E* and are displayed on a logarithmic scale. Dotted line indicates relative effect of one (no effect). *p* values were calculated using Kruskal-Wallis with Dunn’s *post hoc* test of log-transformed values.

Baseline currents produced by mutant ENaC with individual alanine substitutions at critical aromatic α-ENaC residues were significantly reduced (Fig. 4, *B*–*F*) compared to those measured in oocytes expressing WT ENaC (Fig. 4, *A* and *F*). Western blot experiments revealed no substantial reduction in α-ENaC protein expression or cleavage for any of the mutants (Fig. S5), suggesting that the decreased baseline currents likely reflect an inhibitory effect of these mutations on the channel’s P_o_. Indeed, analysis of Na^+^ self-inhibition in these mutants revealed that the steady-state-to-peak current ratio (26, 50, 51) was significantly reduced compared with WT ENaC (Fig. S6). Thus, the reduced currents can, at least in part, be explained by enhanced Na^+^ self-inhibition. Importantly, each alanine substitution markedly attenuated the concentration-dependent inhibition of ENaC by the α-8 peptide (Fig. 4, *A*–*E*, *G*). The effect was most pronounced in the αF226A and αW251A mutants (Fig. 4, *B*, *C* and *G*), which also exhibited the lowest baseline currents (Fig. 4*F*).

Taken together, our findings indicate that the conserved aromatic residues are functionally important in both γ- and α-ENaC and likely mediate interactions with the respective autoinhibitory tracts and synthetic inhibitory peptides.

### Removal of the P1-P2 segment from the GRIP domain of β-ENaC does not alter ENaC currents

Cryo-EM analysis revealed that a structurally similar GRIP domain is also present in β-ENaC (Fig. 1, *A* and *B*). We investigated whether the P1/P2 segment of the GRIP domain in β-ENaC, which corresponds to a channel portion cleaved out by proteases from the α- and γ-subunits, functions as an autoregulatory tract. For this purpose, a thrombin consensus cleavage sequence (VPRGVR; (52, 53)) was introduced into the proximal (after βL131) and/or distal (after βL174) unstructured region flanking the P1/P2 segment. We expected that thrombin would specifically recognize and cleave this sequence, thereby removing the P1/P2 segment. However, for unknown reasons, thrombin cleaved only at the distal site, while the proximal site remained intact (Fig. S7*A*). Consequently, it was not possible to use thrombin to remove the P1/P2 sequence from β-ENaC. We hypothesized that a protease with broader substrate specificity, such as trypsin, could be used instead to cleave the introduced sequence after one of the positively charged arginine residues. However, trypsin, as previously demonstrated (39, 54, 55, 56), would cleave not only β-ENaC but also γ-ENaC at the distal site leading to proteolytic channel activation in functional experiments. To prevent ENaC activation *via* γ-subunit cleavage, we used a mutated γ-ENaC in which the autoinhibitory tract was covalently tethered to its binding site in the thumb domain *via* an introduced disulfide bond (γS155C – γQ426C). This disulfide-locked γ-ENaC variant, resistant to proteolytic activation, was established in our previous study investigating ENaC stimulation by the transmembrane serine protease TMPRSS2 (48). Consistent with our previous observations, the channel containing the disulfide-locked γ-ENaC could not be stimulated proteolytically, unless oocytes were treated with DTT to reduce the disulfide bond (Fig. S8, *A* and *C*). In contrast, DTT treatment had no significant effect on protease-mediated stimulation of WT αβγ-ENaC (Fig. S8, *B* and *C*).

**Figure 5:**
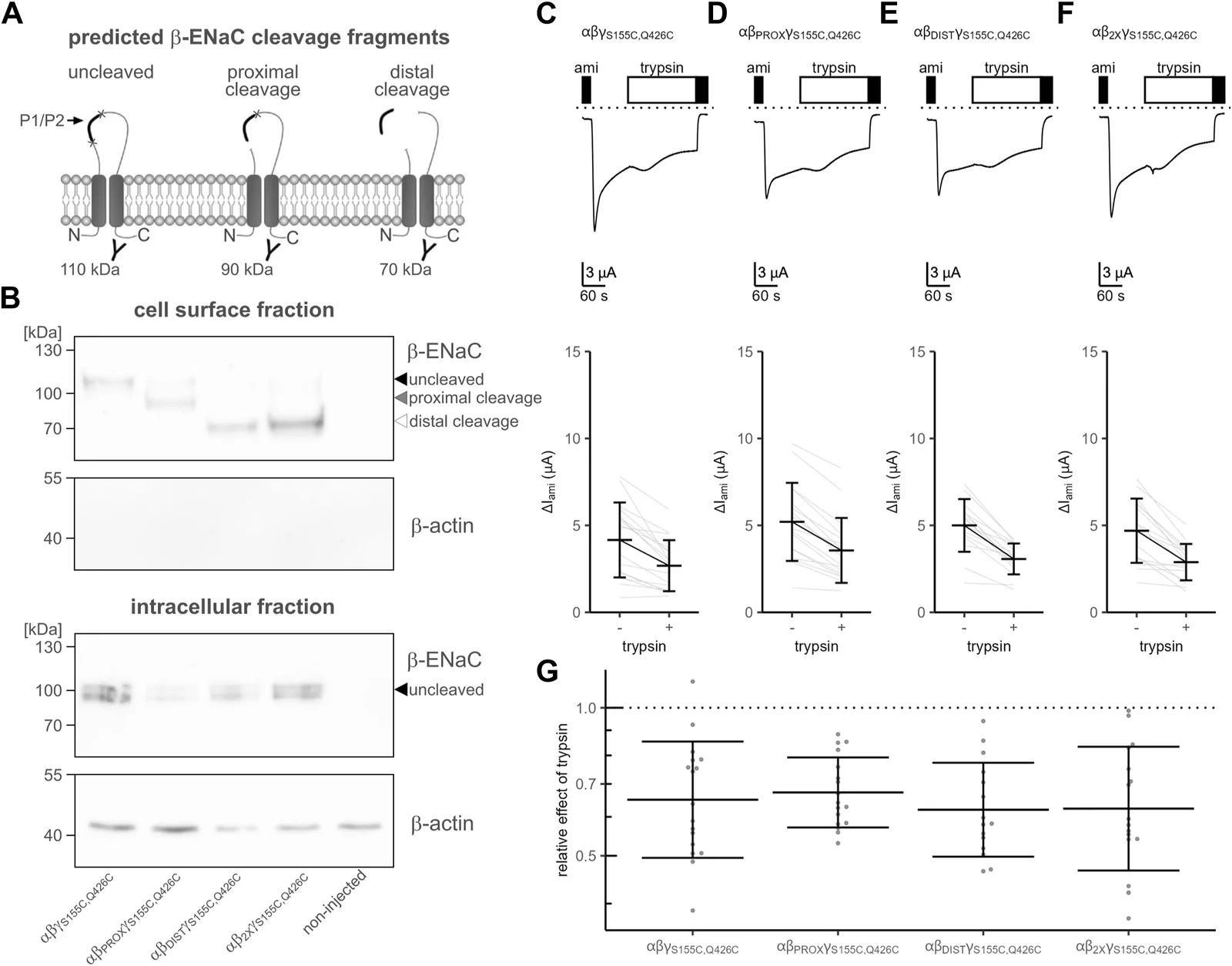
**Proteolytic removal of the P1/P2 segment from the β-subunit does not stimulate ENaC activity.***A*, schematic diagram showing predicted β-ENaC fragments resulting from cleavage at artificially introduced thrombin cleavage sites (depicted as ‘X’) located proximal or distal to the P1/P2 segment, which can be detected using an antibody raised against a C-terminal β-ENaC epitope. The expected molecular weights of the corresponding C-terminal β-ENaC cleavage fragments are given below. *B*, representative Western blots showing cell surface (*upper panel*) or intracellular (*third panel*) expression of β-ENaC in oocytes from one batch co-expressing wild-type α-ENaC (α) and the disulfide-locked γ-ENaC mutant (γ_S155C,Q426C_) together with one of the indicated β-ENaC variants: wild-type (β), with a thrombin cleavage site inserted proximal (β_PROX_ = β_ins131VPRGVR132_), distal (β_DIST_ = β_ins174VPRGVR175_), or both proximal and distal (β_2X_ = β_ins131VPRGVR132, ins174VPRGVR175_) to the P1/P2 segment. Oocytes were incubated in the presence of 2 μg/ml trypsin dissolved in ND96 for 3 min before biotinylation. Specific signal of β-ENaC was detected using an antibody against the C-terminal epitope of β-ENaC. To increase ENaC expression and improve β-ENaC detection in Western blot experiments, oocytes were injected with more than the usual amount of cRNA for ENaC (0.2 ng/subunit/oocyte). Non-injected oocytes served as a control. Uncleaved (∼110 kDa), proximally cleaved (∼90 kDa), and distally cleaved β-ENaC (∼70 kDa) are indicated by *black-filled*, *gray*, and *open* arrowheads, respectively. To validate separation of cell surface proteins from intracellular proteins, blots were stripped and reprobed using an antibody against β-actin (*second and lower panels*). Similar results were obtained in one additional repeat (N = 2). *C-F*, *Upper panels*, A representative whole-cell current trace is shown for an oocyte expressing ENaC constructs as described in (*B*). Amiloride (ami, 2 μM) and trypsin (2 μg/ml) were present in the bath solution as indicated by *black* and *white* bars, respectively. Dotted lines correspond to the zero current level. *Lower panels,* ΔI_ami_ values were determined before (−) and after (+) trypsin application from similar experiments as shown in the *upper panels* as described in Figure 2. *Gray* lines connect data points obtained in an individual oocyte. Mean ± SD are shown in *black* (*C*-*F*: N = 2, 30 ≤ n ≤ 34; N indicates the number of different batches of oocytes, n indicates the number of individual oocytes studied per experimental group). *G*, relative effect of trypsin was calculated for different ENaC constructs using ΔI_ami_ values from the same experiments as shown in *C*-*F* and are displayed on a logarithmic scale. Dotted line indicates relative effect of one (no effect). We observed no statistically significant differences among the four groups, as assessed by one-way ANOVA of log-transformed values (*p* = 0.75).

As expected, incubation of oocytes with trypsin resulted in efficient cleavage of the mutant β-subunits at proximal, distal or both thrombin sites (Fig. 5, *A* and *B*). Proximal cleavage produced a C-terminal β-ENaC fragment of ∼90 kDa, while distal cleavage generated a shorter fragment of ∼70 kDa. In contrast, cleavage was not observed in oocytes expressing WT β-ENaC, where the full-length form of β-ENaC (110 kDa) was detected (Fig. 5, *A* and *B*). Importantly, β-ENaC cleavage by trypsin at the introduced thrombin cleavage sites did not result in proteolytic channel activation when the γ-ENaC-mediated stimulation was prevented by using the disulfide-locked γ-ENaC variant. Thus, by using this disulfide-locked γ-ENaC variant, we were able to demonstrate that β-ENaC cleavage *per se* did not stimulate ENaC activity (Fig. 5, *C*–*G*). Interestingly, in oocytes expressing either WT or mutant β-ENaC, a similar current decline was observed in the presence of trypsin, likely due to non-specific cleavage or spontaneous channel rundown.

In conclusion, our data indicate that the P1/P2 segment of the GRIP domain in the β-subunit does not function as an autoregulatory tract and that its removal does not result in channel activation.

### Proteolytic activation of ENaC in its δβγ-configuration is due to γ- but not δ-ENaC cleavage

It is known from previous studies that human ENaC in its δβγ-configuration can be stimulated by proteases, albeit to a significantly lower degree than αβγ-ENaC (28). However, it remains unclear whether cleavage of the δ-subunit contributes to this proteolytic stimulation, or whether it is mainly mediated by γ-subunit cleavage.

**Figure 6:**
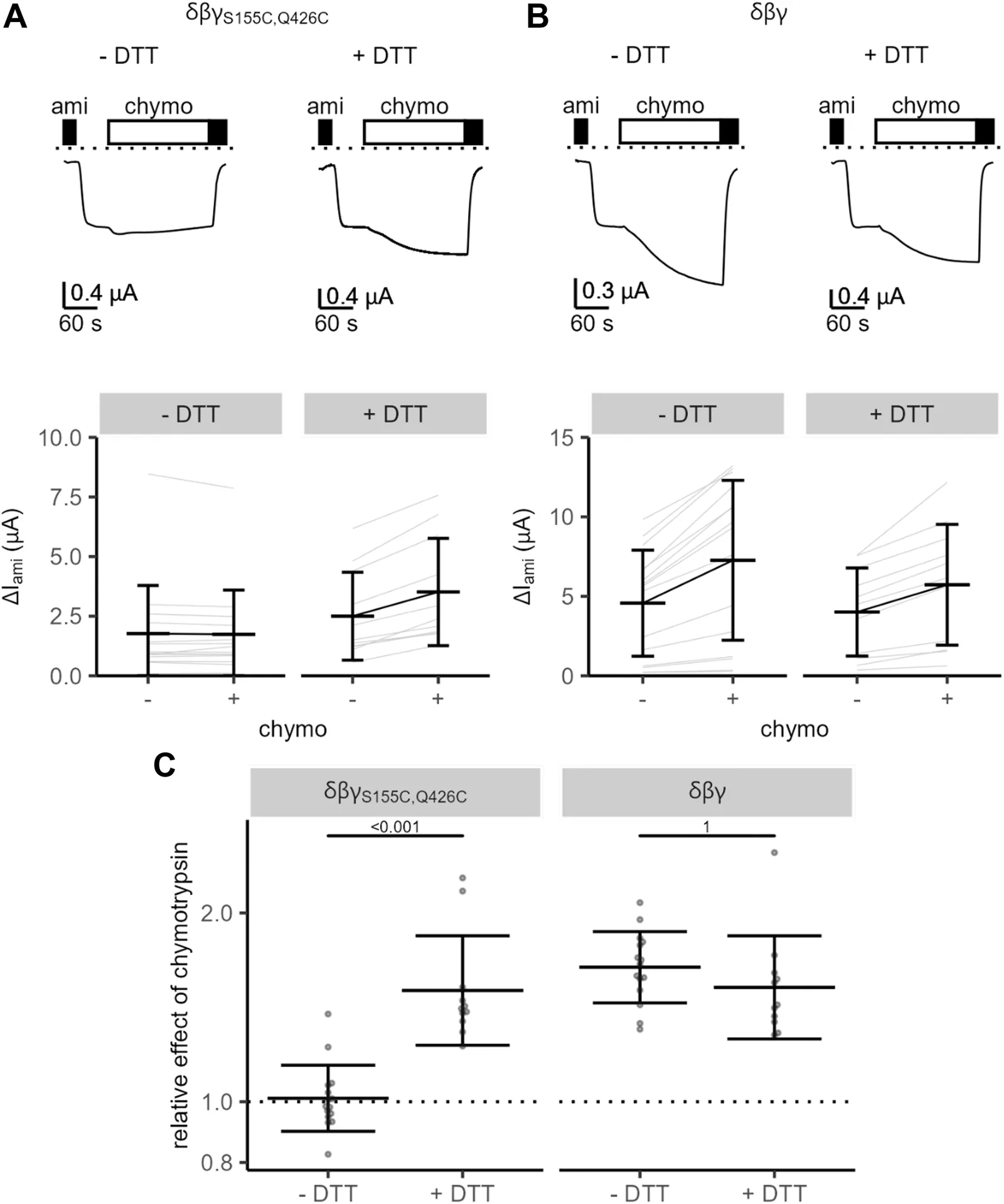
**Preventing the release of the inhibitory tract from γ-ENaC abolishes the stimulatory effect of chymotrypsin on δβγ-ENaC.***A, B*, *upper panels*, A representative whole-cell current trace is shown for an oocyte co-expressing human wild-type δ- and β-ENaC together with either the disulfide-locked γ-ENaC mutant (δβγ_S155C,Q426C_, *A*) or the wild-type γ-subunit (δβγ; *B*). Oocytes were incubated either in ND9 without DTT (−DTT, *left panels*) or with 30 mM DTT (+DTT, *right panels*) for 15 min before current measurement. Amiloride (ami, 100 μM) and chymotrypsin (2 μg/ml) were present in the bath solution as indicated by *black* and *white* bars, respectively. Dotted lines correspond to the zero current level. *Lower panels*, ΔI_ami_ values were determined before (−) and after (+) chymotrypsin application from similar experiments as shown in the *upper panels* as described in Figure 2. *Gray* lines connect data points obtained in an individual oocyte. Mean ± SD are shown in black (*A*, *B*: N = 2, n = 26; N indicates the number of different batches of oocytes, n indicates the number of individual oocytes studied per experimental group). *C,* Relative stimulatory effect of chymotrypsin was determined using ΔI_ami_ values from the same experiments as shown in *A*, *B* and are displayed on a logarithmic scale. Dotted line indicates relative effect of one (no effect). *p* values were calculated using one-way ANOVA with Bonferroni *post hoc* test of log-transformed values.

To investigate the potential contribution of δ-ENaC to proteolytic channel stimulation, we employed a strategy analogous to that described above for β-ENaC: we co-expressed WT δ- and β-ENaC with the disulfide-locked γ-ENaC mutant (γS155C, γQ426C) resistant to proteolytic activation. Importantly, this channel configuration could not be stimulated by chymotrypsin (Fig. 6, *A* and *C*). Treatment of oocytes expressing this mutant channel with DTT to reduce the disulfide bond restored the typical ∼1.8-fold stimulatory effect of chymotrypsin (Fig. 6, *A* and *C*), similar to that observed in oocytes expressing WT δβγ-ENaC (Fig. 6, *B* and *C*). In contrast, DTT produced no significant effect on ENaC lacking the disulfide bond in the γ-subunit (Fig. 6, *B* and *C*). In conclusion, these findings strongly suggest that δβγ-ENaC is stimulated by proteases *via* cleavage of the γ-subunit, whereas δ-ENaC cleavage does not contribute to proteolytic channel activation.

**Figure 7:**
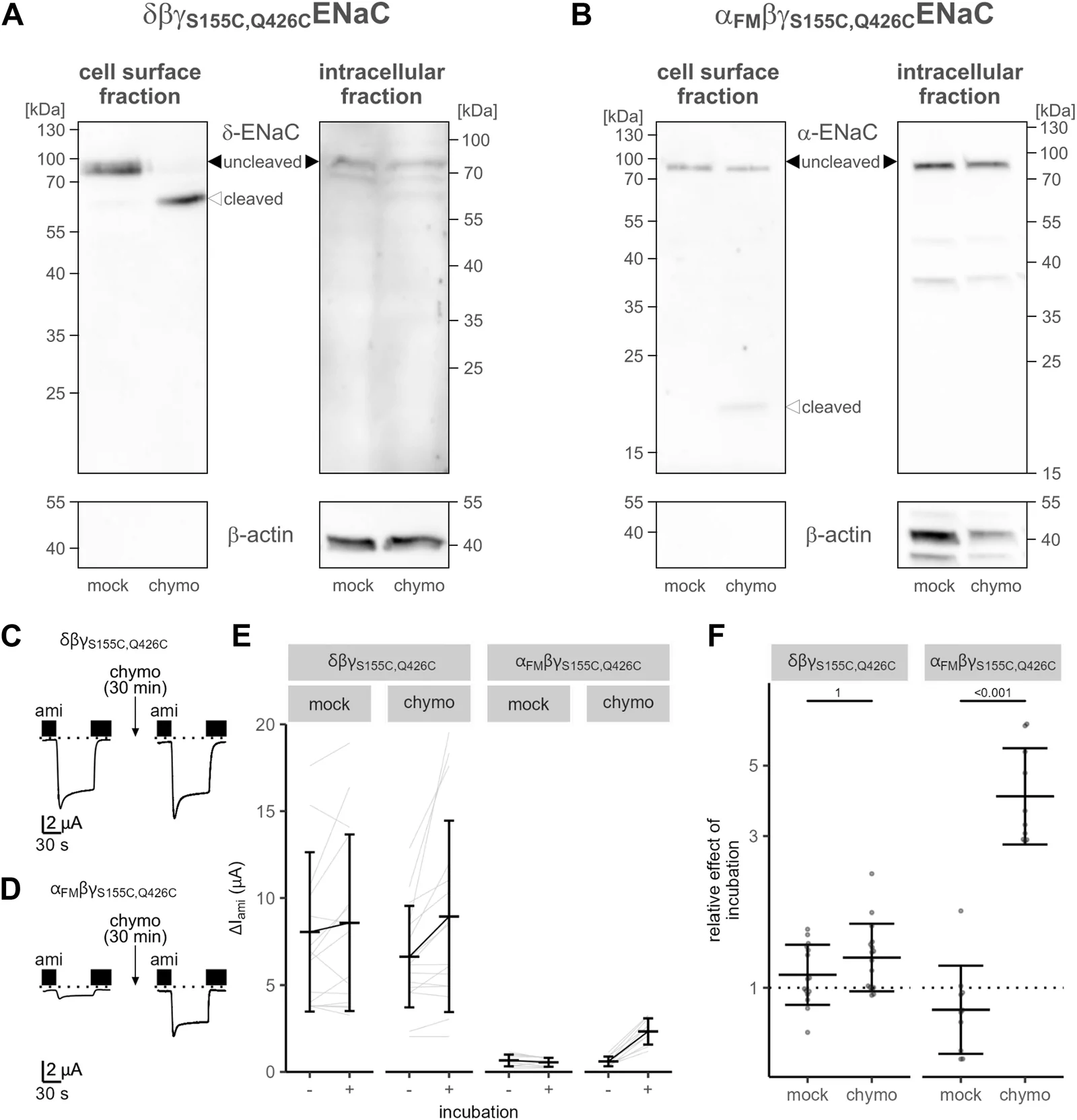
**In contrast to α-ENaC, proteolytic cleavage of δ-ENaC does not lead to a substantial stimulation of ENaC-mediated currents.***A*, *B*, representative Western blots showing cell surface (*upper left panels*) or intracellular (*upper right panels*) expression of δ-ENaC (*A*) or α-ENaC (*B*) in oocytes from one batch co-expressing wild-type β-ENaC (β) and the disulfide-locked γ-ENaC mutant (γ_S155C,Q426C_) together with either wild-type δ-ENaC (δ, *A*) or the α-ENaC mutant in which both proximal (_175_RSRR_178_) and distal (_201_RRAR_204_) furin cleavage sites were mutated to alanines (α_FM_ = _175_RSRR_178_-AAAA, _201_RRAR_204_-AAAA, *B*). Oocytes were incubated either in mock solution (ND96 supplemented with 100 μM or 2 μM amiloride for δβγ_S155C,Q426C_ or α_FM_βγ_S155C,Q426C_, respectively) or in the presence of 2 μg/ml chymotrypsin (also in ND96 supplemented with 100 μM or 2 μM amiloride for δβγ_S155C,Q426C_ or α_FM_βγ_S155C,Q426C_, respectively) for 30 min before biotinylation. Specific signals of δ-ENaC and α-ENaC were detected using an antibody against the C-terminal epitope of δ-ENaC and N-terminal epitope of α-ENaC, respectively. To increase ENaC expression and improve detection of δ- and α-ENaC in Western blot experiments, oocytes were injected with more than the usual amount of cRNA for ENaC (0.5 ng/subunit/oocyte). Uncleaved δ- and α-ENaC (∼90 kDa) are indicated by *black-filled* arrowheads. Cleaved δ- (∼65 kDa) and α-ENaC (∼20 kDa) are indicated by *open* arrowheads. Original uncropped images of these Western blots are shown in Fig. S9 to demonstrate the specificity of the antibodies against δ- and α-ENaC. To validate separation of cell surface proteins from intracellular proteins, blots were stripped and reprobed using an antibody against β-actin (*lower panels*). Similar results were obtained in one additional repeat (N = 2). ΔI_ami_ measurements were performed in parallel in oocytes from the same batch (*C*-*F*). *C, D*, a representative whole-cell current trace is shown for an oocyte expressing δβγ_S155C,Q426C_ (*C*) or α_FM_βγ_S155C,Q426C_ (*D*) before (*left traces*) and after 30 min of chymotrypsin treatment (*right traces*). Amiloride (ami; 100 μM in *C* or 2 μM in *D*) was present in the bath solution as indicated by *black* bars. Dotted lines correspond to the zero current level. *E*, ΔI_ami_ values were determined as described in Figure 2 in chymotrypsin-treated oocytes as shown in *C*, *D* and additionally in mock-treated oocytes before (−) and after (+) correspondent incubation. Incubation conditions were the same as described in *A*, *B*. *Gray* lines connect data points obtained in an individual oocyte. Mean ± SD are shown in *black* (N = 2, 10 ≤ n ≤ 16; N indicates the number of different batches of oocytes, n indicates the number of individual oocytes studied per experimental group). *F*, relative effect of mock or chymotrypsin incubation on ΔI_ami_ obtained from the same experiments as shown in *E* and displayed on a logarithmic scale. Dotted line indicates relative effect of one (no effect). *p* values were calculated using Kruskal-Wallis with Dunn’s *post hoc* test of log-transformed values.

In an additional set of experiments, we injected an increased amount of cRNA to improve Western blot detection of δ-ENaC. Consistent with our earlier observations (28), we observed no proteolytic processing of the δ-subunit by intracellular furin-like convertases and detected only full-length δ-ENaC at ∼90 kDa. This is in line with the absence of canonical furin consensus motifs in the δ-ENaC sequence. Importantly, we demonstrated that a 30-min treatment with chymotrypsin resulted in cleavage of δ-ENaC at the cell surface, evidenced by a smaller fragment of ∼65 kDa (Figs. 7*A* and S9*A*). However, no substantial chymotrypsin-induced stimulation of ENaC currents, measured in parallel with protein detection, was observed (Fig. 7, *C*, *E* and *F*), because the disulfide-locked γ-ENaC mutant prevented proteolytic activation of the channel through the γ-subunit. In control experiments performed using the same protocol, a typical two-fold stimulatory effect of chymotrypsin on WT δβγ-ENaC was observed (Fig. S10, *A*, *C* and *D*). Thus, consistent with the results described above, δβγ-ENaC is proteolytically stimulated *via* γ- but not δ-ENaC cleavage.

Importantly, using the disulfide-locked γ-ENaC mutant we could clearly demonstrate that α-ENaC, in contrast to δ-ENaC, contributes to proteolytic stimulation of αβγ-ENaC. In the experiments shown in Figure 7, endogenous furin-mediated removal of the α-autoinhibitory tract was prevented by introducing specific mutations in the α-subunit (α_FM_ = _175_RSRR_178_-AAAA, _201_RRAR_204_-AAAA). The α-tract could then be released by exogenously applied chymotrypsin. Treatment of oocytes expressing α_FM_βγ_S155C,Q426C_-ENaC with chymotrypsin caused α-ENaC cleavage and an approximately four-fold increase in currents (Fig. 7, *B*, *D*–*F*). In control experiments with WT γ-ENaC instead of the disulfide-locked mutant, a much stronger (∼20-fold) chymotrypsin-mediated activation of ENaC currents was observed (Fig. S10, *B*–*D*), indicating that the contribution of the γ-subunit is greater than that of the α-subunit. It should be noted that proteolytic processing of the mutant α-ENaC by chymotrypsin was not complete under our experimental conditions (Figs. 7*B* and S9B). This may result in an underestimation of the relative contribution of α-ENaC cleavage to proteolytic channel activation. Nonetheless, our findings clearly allow the conclusion that α-cleavage significantly contributes to proteolytic channel activation, whereas cleavage of the δ-subunit does not contribute to proteolytic ENaC activation.

Finally, it is worth pointing out that replacing uncleaved α_FM_-ENaC with δ-ENaC dramatically increased baseline ENaC currents (Fig. S10, *A*–*C*) and substantially decreased the relative stimulatory effect of chymotrypsin (Fig. S10, *A*, *B* and *D*) consistent with our previous report (28). This finding supports the concept that δ-ENaC, although not directly contributing to proteolytic channel activation, induces conformational rearrangements that enhance baseline activity of ENaC.

### Removal of the P1 segment from the GRIP domain of δ-ENaC does not alter ENaC currents

Notably, the Western blot results shown in Figure 7*A* do not clarify whether chymotrypsin completely removed the P1 segment from δ-ENaC. To directly test whether the P1 segment in the δ-subunit functions as an autoregulatory tract, we adopted a strategy similar to that used for β-ENaC, generating δ-ENaC constructs harboring thrombin cleavage sites flanking the P1 segment. However, this δ-ENaC construct was not cleaved by thrombin at the introduced cleavage sites (Fig. S7*B*). Therefore, like in the case of β-ENaC, we applied trypsin instead of thrombin to cleave both thrombin sites in this δ-ENaC construct. To prevent concomitant proteolytic activation of γ-ENaC by trypsin in these experiments, we used the disulfide-locked γ-ENaC mutant (γS155C, γQ426C). This approach allowed us to test specifically the effect of δ-ENaC proteolysis by exogenously applied trypsin, ensuring cleavage both proximal and distal to the P1 tract of δ-ENaC.

**Figure 8:**
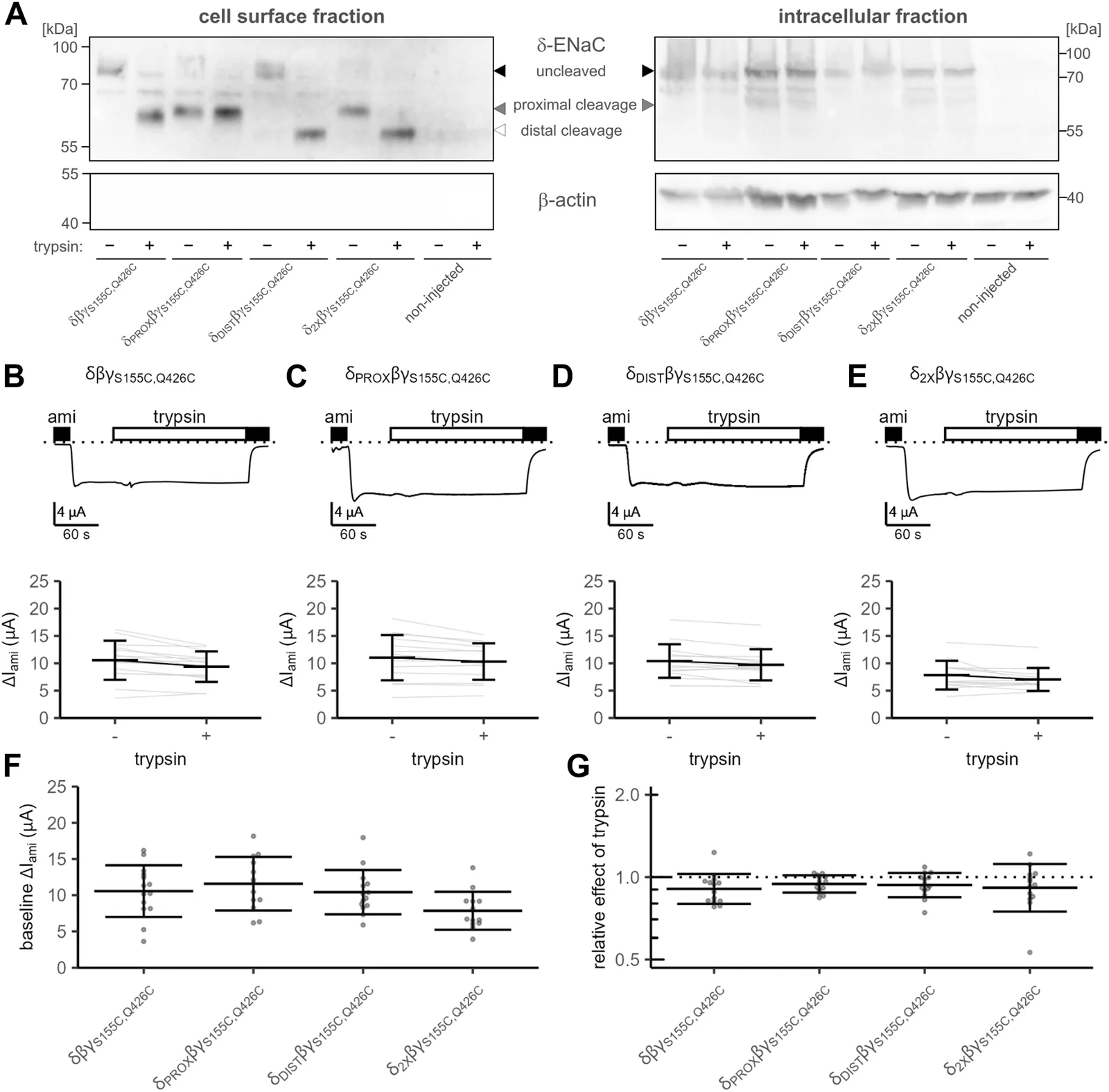
**Proteolytic removal of the P1 segment from the δ-subunit does not stimulate ENaC activity.***A,* representative Western blots showing cell surface (*left upper panel*) or intracellular (*right upper panel*) expression of δ-ENaC in oocytes from one batch co-expressing wild-type β-ENaC (β), the disulfide-locked γ-ENaC mutant (γ_S155C,Q426C_), together with one of the indicated δ-ENaC variants: wild-type (δ), with a thrombin cleavage site inserted proximal (δ_PROX_ = δ_ins170LVPRGV171_), distal (δ_DIST_ = δ_ins199LVPRGVR200_), or both proximal and distal (δ_2X_ = δ_ins170LVPRGV171, ins199LVPRGVR200_) to the P1 segment. Similar to our experiments with mutated β-ENaC (Fig. 5), oocytes were incubated in the presence of 2 μg/ml trypsin dissolved in ND96 for 3 min before biotinylation. Specific signal of δ-ENaC was detected using an antibody against the C-terminal epitope of δ-ENaC. Non-injected oocytes served as a control. Uncleaved (∼90 kDa), proximally cleaved (∼65 kDa), and distally cleaved δ-ENaC (∼60 kDa) are indicated by *black-filled*, *gray*, and *open* arrowheads, respectively. To validate separation of cell surface proteins from intracellular proteins, blots were stripped and reprobed using an antibody against β-actin (*lower panels*). Similar results were obtained in one additional repeat (N = 2). *B-E*, *Upper panels*, A representative whole-cell current trace is shown for an oocyte expressing ENaC constructs as described in *A*. Amiloride (ami, 2 μM) and trypsin (2 μg/ml) were present in the bath solution as indicated by *black* and *white* bars, respectively. Dotted lines correspond to the zero current level. *Lower panels,* ΔI_ami_ values were determined before (−) and after (+) trypsin application from similar experiments as shown in the *upper panels* as described in Figure 2. *Gray* lines connect data points obtained in an individual oocyte. Mean ± SD are shown in black (*B-E*: N = 1, n = 14; N indicates the number of different batches of oocytes, n indicates the number of individual oocytes studied per experimental group). *F*, Baseline ΔI_ami_ values before application of trypsin were obtained for different ENaC constructs in the same experiments as shown in *B*–*E*. *G*, Relative effect of trypsin was calculated for different ENaC constructs using ΔI_ami_ values from the same experiments as shown in *B*–*E* and are displayed on a logarithmic scale. Dotted line indicates relative effect of one (no effect). We observed no statistically significant differences among the four groups, as assessed by Kruskal-Wallis test (*F*: *p* = 0.16; *G*: values were log-transformed before statistical testing, *p* = 0.55).

Consistent with our experiments using chymotrypsin (Fig. 7*A*), trypsin treatment shifted the δ-ENaC signal from ∼90 kDa to ∼65 kDa (Fig. 8*A*). Surprisingly, δ-ENaC with an introduced proximal cleavage site (δ_PROX_) migrated at ∼65 kDa even without trypsin treatment, suggesting cleavage by an endogenous protease in *X. laevis* oocytes. Indeed, this ∼65 kDa fragment was also detected in the intracellular fraction alongside the ∼90 kDa full-length band, indicating that the δ_PROX_ mutant was cleaved by an intracellular protease. Trypsin treatment did not significantly alter the cleavage pattern of δ_PROX_. Taken together, these findings suggest that trypsin and chymotrypsin likely target a region proximal to the P1 tract in the WT sequence, producing only a single cleavage event.

Importantly, δ-ENaC carrying a distal engineered cleavage site (δ_DIST_) migrated at ∼90 kDa before and at ∼60 kDa after trypsin treatment, consistent with selective processing at the introduced distal site. This additional distal processing by trypsin was further confirmed using the δ-ENaC mutant containing both proximal and distal cleavage sites (δ_2X_), in which trypsin shifted the δ-ENaC fragment from ∼65 kDa to ∼60 kDa. Collectively, these data demonstrate that the introduction of engineered cleavage sites enables complete removal of the P1 segment from δ-ENaC.

To determine whether this proteolytic processing translates into functional effects, we measured ENaC-mediated currents in all δβγ-ENaC constructs before and after trypsin treatment (Fig. 8, *B*–*H*). Baseline currents were similar across constructs. Importantly, trypsin did not substantially alter current levels in any of the tested δβγ-ENaC heterotrimers when activation through the γ-subunit was prevented by the introduced disulfide bond. Thus, despite clear evidence of proteolytic processing of δ-ENaC with cleavage occurring proximally and distally of the P1 tract, the resulting removal of this tract does not confer functional effects.

## Discussion

In the present study, we conducted a systematic functional analysis of the GRIP domains in human αβγ- and δβγ-ENaC, guided by MD simulations based on available structural information on ENaC. Additionally, we investigated whether cleavage of the δ-subunit contributes to the proteolytical activation of human δβγ-ENaC. The key observations are as follows: 1) conserved aromatic residues (Phe204, Trp229, His233, and Tyr425) that form the binding site for the P1 segment of the GRIP domain in γ-ENaC are essential for channel inhibition by both the γ-autoinhibitory tract and the synthetic γ-11 peptide; 2) conserved aromatic residues (Phe226, Trp251, His255, and Tyr447) that form the binding site for the P1 segment of the GRIP domain in α-ENaC are critically important for channel inhibition by the synthetic α-8 peptide; 3) the GRIP domains of β- and δ-ENaC do not contain corresponding autoregulatory tracts; 4) proteolytic activation of δβγ-ENaC is mediated by cleavage of the γ-subunit, but not the δ-subunit. In conclusion, our work provides new mechanistic insights into the proteolytic activation of ENaC.

In this study, we experimentally validated several critical features of the available structural model of αβγ-ENaC (33, 34). In particular, our MD simulations, performed using the published cryo-EM structure of αβγ-ENaC (34), predicted that four conserved aromatic residues (Phe204, Trp229, His233, and Tyr425 in γ-ENaC; Phe226, Trp251, His255, and Tyr447 in α-ENaC) form particularly stable interactions with the P1 segment of the GRIP domain in the corresponding subunit. These P1 segments correspond to critical regions of the γ- and α-autoinhibitory tracts, which are cleaved by proteases to stimulate channel activity (44, 45). Therefore, replacing these aromatic residues with the small nonpolar alanine in uncleaved γ-ENaC was expected to disturb side-chain interactions between the γ-autoinhibitory tract and its binding site, thereby stimulating ENaC without actual cleavage of the γ-subunit. Indeed, the ENaC mutants exhibited significantly increased baseline currents and reduced (F204A, H233A) or even abolished (W229A, Y425A) the stimulatory effect of chymotrypsin, indicating that these mutations mimic proteolytic channel activation. Furthermore, similar effects were observed when key interaction partners of the aromatic cluster within the P1 segment, particularly Ile158 and Leu160, were substituted with alanine. This finding is consistent with previous work demonstrating the important role of Ile and Leu residues in mouse ENaC inhibition by a synthetic γ-11 peptide (45). The observation that mutating critical residues either in the P1 segment or in its binding site leads to ENaC stimulation validates structural data and supports our hypothesis that increased ENaC activity observed in these mutants results from disruption of specific interactions between the autoinhibitory P1 segment and its binding site.

However, we cannot exclude the possibility that the mutations tested induce additional structural changes in ENaC, which for example may affect Na^+^ self-inhibition of the channel. Na^+^ self-inhibition is an intrinsic regulatory mechanism of ENaC by which elevated extracellular Na^+^ reduces channel P_o_ (50, 51, 57, 58). Interestingly, it is mechanistically linked to proteolytic channel activation (51, 58), and a naturally occurring gain-of-function mutation in human α-ENaC (W493R) mimicking proteolytic channel activation reduces Na^+^ self-inhibition (26). Previous studies highlighted that the stimulatory effect of both α- and γ-ENaC cleavage events relies, at least in part, on relieving Na^+^ self-inhibition (51, 58, 59). Moreover, it was shown that mutation of Trp235 and His239 in mouse γ-ENaC (analogous to Trp229 and His233 in human γ-ENaC) essentially abolishes Na^+^ self-inhibition (50, 57). Therefore, the increased baseline currents of our P1 binding site mutants in the γ-subunit may reflect reduced Na^+^ self-inhibition, possibly caused by the impaired interaction between the γ-autoinhibitory tract and its binding pocket.

Importantly, the binding site mutations substantially reduced the inhibitory effect of the synthetic γ-11 peptide, indicating that γ-11 and the autoinhibitory tract act through the same binding site in human γ-ENaC. This is consistent with previous findings in the mouse ortholog (45, 60). Specifically, Balchak *et al.* demonstrated that the N-terminus of the synthetic peptide can be chemically cross-linked to a cysteine introduced at Gln426 (Q426C), a residue adjacent to the critical Tyr425 identified in the present study (60). Notably, an increased P_o_ has been reported to reduce the apparent affinity of γ-11 for ENaC (45). In our experiments, γ-11 was applied after stimulation of ENaC with chymotrypsin, which raises the P_o_ of WT ENaC to near unity (28, 39, 48, 49). Following chymotrypsin treatment, ENaC-mediated currents reached similar levels across WT and mutant channels, suggesting that P_o_ was comparably high in all ENaC variants prior to γ-11 application. Therefore, differences in P_o_ are unlikely to account for the reduced inhibitory effect of the γ-11 peptide observed in the binding site mutants in the present study.

Interestingly, previous work has shown that the inhibitory effect of γ-11 is largely preserved in the presence of the γ-autoinhibitory tract (45). This suggests that the endogenous inhibitory tract does not fully prevent access of the synthetic peptide to the inhibitory binding site, in particular when the local γ-11 concentration is high. Moreover, cleavage at the proximal cleavage site may enhance the mobility of the endogenous γ-autoinhibitory tract. Interestingly, in high concentrations γ-11 can cause almost complete ENaC inhibition and thus appears to inhibit the channel more effectively than the endogenous γ-inhibitory tract itself (45). Further studies are needed to elucidate the precise mechanism by which the γ-11 peptide exerts its effect on uncleaved γ-ENaC containing the intact autoinhibitory tract. For example, we cannot exclude the possibility that γ-11 partially exerts its inhibitory effect by binding to a structurally similar site in the α-subunit, particularly at high concentrations (>10 μM). However, the strong reduction of γ-11 inhibition caused by point mutations (F204A, W229A, H233A, and Y425A) indicates that, at the concentrations used here (1 and 10 μM), the inhibitory effect of γ-11 on ENaC is primarily mediated by binding to the corresponding inhibitory binding site in the γ-subunit.

In the α-subunit, mutation of the conserved aromatic residues (F226A, W251A, H255A, Y447A) strongly reduced channel inhibition by the synthetic α-8 peptide, indicating that α-8 acts through the P1 binding site in the α-subunit. A previous systematic tryptophan-scanning mutagenesis in mouse α-ENaC similarly identified the corresponding histidine residue as functionally important (H282W) for inhibition by the α-8 peptide, whereas the F253W and Y474W mutations (corresponding to human Phe226 and Tyr447) did not significantly alter the blocking effect of the α-8 peptide (61). These apparent discrepancies may arise from the use of tryptophan substitutions in those experiments. Because tryptophan is an aromatic residue, unlike the alanine used in our study, it is less likely to disrupt the aromatic cluster critical for the α-8 binding. In a follow-up study, Kashlan *et al.* found that the Y474C mutant spontaneously forms a disulfide bond with a cysteine residue introduced at the N-terminus of the α-8 peptide (62). This finding supports a direct interaction between Y474 and the inhibitory peptide, consistent with both structural information on ENaC and our functional data. Although this was not assessed experimentally, we assume that the aromatic residues are also important for the interaction of the endogenous α-autoinhibitory tract with α-ENaC. This conclusion is consistent with our previous observation that the α-inhibitory peptide requires an intact Leu188 residue, the key interaction partner of the aromatic Trp251 residue, to produce its inhibitory effect on ENaC (46).

Notably, all tested α-ENaC mutants, especially F226A and W251A, demonstrated substantially reduced baseline currents, which is probably due to reduced channel P_o_ and complicates the interpretation of our findings. For example, the introduced mutations may inhibit the channel by causing similar structural rearrangements as α-8 binding. Moreover, it was previously shown that mutations of His282 in mouse α-ENaC (equivalent to human His255) enhanced Na^+^ self-inhibition and reduced macroscopic currents (50), providing another plausible explanation for the reduced baseline currents we observed in the H255A mutant. Consistent with these earlier findings, we confirmed that H255A mutation significantly enhanced Na^+^ self-inhibition. In addition, we demonstrated that the F226A, W251A, and Y447A mutations likewise significantly increased Na^+^ self-inhibition. Interestingly, the cation binding site, which may serve as a Na^+^ sensor to mediate ENaC inhibition by extracellular Na^+^, is formed by the β6-β7 loop localized in close vicinity to the P1 binding site in the α-subunit (34, 63). Therefore, it is plausible that the P1 binding site mutations may facilitate interactions of Na^+^ with the cation binding site and thus enhance Na^+^ self-inhibition. In a previous study it was demonstrated that the apparent affinity of the α-peptide inversely correlated with the P_o_ of ENaC (61). Thus, it is unlikely that the reduced P_o_ of the P1 binding site mutants in α-ENaC explains the attenuated inhibition by α-8. Taken together, our study provides evidence that the binding sites for the P1 segments of the GRIP domains in γ- and α-ENaC, formed by portions of the finger, GRIP and thumb domains, mediate channel inhibition by the respective autoinhibitory tracts and synthetic inhibitory peptides.

Identification and characterization of the specific inhibitory binding sites within the γ- and α-subunits may represent an essential first step toward the structure-guided development of novel peptidomimetic modulators of ENaC. Currently, only two drugs that directly target ENaC, triamterene and amiloride, are approved for clinical use. Particularly when combined with other antihypertensive agents, these drugs can be used effectively to treat resistant arterial hypertension (25, 64, 65, 66). However, these compounds lack high specificity for ENaC (20, 67) and exhibit poor pharmacokinetic properties for targeting ENaC in the lung (23, 24). Furthermore, as positively charged pore blockers, they may compete with permeating sodium ions for the same binding site, resulting in variable potency that depends on extracellular sodium concentration (4). Therefore, the development of novel peptidomimetic ENaC inhibitors, based on the knowledge of the binding sites in α- and/or γ-ENaC, may offer a promising strategy for designing ENaC-targeting drugs with improved pharmacological profiles (68).

It is well established that β-ENaC is not cleaved by either intracellular or extracellular proteases and does not contribute to proteolytic ENaC activation (36). Striking structural similarities between the GRIP domain of β-ENaC and those of α- and γ-ENaC, however, prompted us to test the hypothesis that the P1/P2 segment of β-GRIP functions as an autoregulatory tract. For this purpose, we designed a β-ENaC mutant containing two artificial cleavage sites flanking the P1/P2 segment. Using this construct, it was possible to remove this segment proteolytically with trypsin. Importantly, to study the effect of β-ENaC cleavage independent of γ-ENaC cleavage, we used a γ-ENaC mutant in which the γ-autoinhibitory tract was tethered to its binding site *via* a disulfide bond. Under non-reducing conditions, proteases can cleave γ-ENaC but elicit no stimulatory effect because the γ-autoinhibitory tract remains attached to the channel. Using essentially the same strategy, we previously showed that the transmembrane protease TMPRSS2 activates αβγ-ENaC by releasing the autoinhibitory tract from γ-ENaC (48). Importantly, β-ENaC cleavage produced no stimulatory effect on ENaC activity. In conclusion, our data indicate that the P1/P2 segment of the GRIP domain in β-ENaC plays no autoregulatory role. This is consistent with cryo-EM data on ENaC indicating that β-ENaC provides a structural scaffold within the heterotrimeric ENaC assemblies (35). Interestingly, we have recently demonstrated that the small molecule ENaC activator S3969 stimulates ENaC by interacting with a specific binding site within the β-subunit (47, 69). Moreover, this stimulatory effect appears to be additive to that produced by proteases in airway epithelial H441 cells (48, 56, 69). Therefore, while β-ENaC does not contribute to proteolytic ENaC activation, it appears to play a critical role in ligand-mediated ENaC regulation.

In a previous study, we demonstrated that human ENaC in its δβγ-configuration can be activated by chymotrypsin in *Xenopus leavis* oocytes, and that this stimulatory effect is accompanied by the appearance of δ-ENaC cleavage fragments (28). However, it remained unclear whether δ-ENaC cleavage contributes to chymotrypsin-mediated stimulation of δβγ-ENaC. The presence of a structurally similar GRIP domain in δ-ENaC suggests its potential involvement in channel activation by proteases. Importantly, in the present work we demonstrated that δβγ-ENaC is proteolytically stimulated due to cleavage of the γ-subunit, but not the δ-subunit. Indeed, the δβγ-ENaC mutant in which the γ-autoinhibitory tract was tethered to its binding site *via* a disulfide bond could not be activated by chymotrypsin unless DTT was applied to reduce the disulfide bond. In Western blot experiments, we found that δ-ENaC was effectively cleaved by chymotrypsin, indicating that the lack of chymotrypsin-mediated stimulation was not due to the absence of δ-ENaC cleavage. Furthermore, by introducing artificial cleavage sites proximal and distal to the P1 segment to enable its complete proteolytic removal, we confirmed that it does not function as an autoregulatory tract. Our biochemical experiments also indicated that both chymotrypsin and trypsin cleaved wild-type δ-ENaC only once, at a site proximal to the P1 region, where potential cleavage sites for chymotrypsin (*e.g.*, phenylalanine) and trypsin (*e.g.*, arginine) are located. However, this cleavage does not appear to play any functional role in proteolytic channel activation. In conclusion, our findings indicate that the δ-subunit, despite possessing a structurally similar GRIP domain, does not contribute to the proteolytic activation of δβγ-ENaC.

In addition, we confirmed the well-established role of the α-subunit in proteolytic channel activation (37). Furthermore, our results are consistent with previous observations indicating that the γ-subunit is particularly important for the proteolytic activation of αβγ-ENaC (39, 70). Thus, the γ-subunit plays a dominant role in proteolytic ENaC activation not only in the channel’s αβγ- (70) but also in its δβγ-configuration. We also confirmed previous findings that replacing the α-subunit with the δ-subunit markedly increased baseline currents and substantially reduced the relative stimulatory effect of extracellular chymotrypsin (28, 30). In other species, such as *X. laevis* (32) and guinea pig (30, 71), substitution of α-ENaC with δ-ENaC completely abolished ENaC activation by extracellular proteases. Interestingly, a recent structural study revealed that substitution of the α-subunit with the δ-subunit induces significant conformational rearrangements of the channel (35). In particular, δβγ-ENaC exhibits an altered conformation of the finger and GRIP domains in the γ-subunit compared with αβγ-ENaC. These structural changes may underlie the reduced protease sensitivity of δβγ-ENaC, given the critical role of the finger and GRIP domains in proteolytic channel activation.

Collectively, the findings of the present study improve our understanding of the molecular mechanisms involved in proteolytic ENaC activation. In particular, they indicate that, unlike the α- and γ-subunits, the β- and δ-subunits do not contain autoregulatory tracts within their GRIP domains. Furthermore, δ-ENaC does not contribute to proteolytic activation of δβγ-ENaC, which is mediated by cleavage of the γ-subunit. The synthetic γ-11 and α-8 inhibitory peptides likely act *via* the same binding sites that are occupied by the corresponding autoinhibitory tracts in the uncleaved γ- and α-subunits. Identification of key aromatic residues within these binding sites may facilitate the development of novel peptidomimetic ENaC inhibitors with potential (patho)physiological relevance.

## Experimental procedures

### Chemicals

α-Chymotrypsin Type I, trypsin from bovine pancreas, thrombin from bovine plasma, dithiothreitol (DTT), and amiloride were obtained from Sigma-Aldrich. The ENaC α-8- and γ-11-inhibitory peptides (α-8: LPHPLQRL; γ-11: RFSHRIPLLIF) were synthesized by ThermoFisher.

### Plasmids

Full-length complementary DNAs (cDNAs) encoding human α-, β-, and γ-ENaC were kindly provided by H. Cuppens (Leuven, Belgium), while a cDNA encoding human δ-ENaC was provided by R. Waldmann (Valbonne, France). All cDNAs were subcloned into the pGEM-HE vector for heterologous expression in *X. laevis* oocytes. The plasmids were linearized and used as templates for cRNA synthesis with T7 RNA polymerase (mMessage mMachine, Ambion). Mutations in ENaC subunits were introduced using the QuikChange Lightning site-directed mutagenesis kit (Agilent Technologies). Sequence verification was routinely performed through sequence analysis (LGC Genomics).

### Isolation of oocytes and two-electrode voltage clamp (TEVC) experiments

Ovarian lobes were removed *via* partial ovariectomy under anesthesia with Tricain 0.2%, following the principles of German regulations, with approval from the animal welfare officer at the University of Erlangen-Nürnberg (FAU), and under the oversight of the state veterinary health inspectorate. Oocytes were isolated from the ovarian lobes using type-2 collagenase from *Clostridium histolyticum* (Sigma-Aldrich). Unless specified otherwise, defolliculated stage V-VI oocytes were routinely injected with 0.03 ng of cRNA per ENaC subunit (α or δ, β, and γ). After injection, oocytes were incubated in a low-sodium ND9 solution (composition in millimolar: 9 NaCl, 2 KCl, 87 N-methyl-D-glutamine-Cl, 1.8 CaCl_2_, 1 MgCl_2_, 5 HEPES, pH 7.4 adjusted with Tris) supplemented with 100 units/ml sodium penicillin and 100 μg/ml streptomycin sulfate.

Two-electrode voltage clamp (TEVC) measurements were conducted 48 h after cRNA injection, essentially as described previously (48, 72, 73, 74, 75). For TEVC experiments, single oocytes were placed in a flow chamber and were superfused with a corresponding bath solution at a flow rate of approximately 2 ml/min. ENaC-mediated whole-cell currents (ΔI_ami_) were measured by washing out amiloride (2 μM for αβγ-ENaC or 100 μM for δβγ-ENaC) with an amiloride-free bath solution and subtracting the averaged whole-cell currents recorded without amiloride from the currents measured in the presence of amiloride at the beginning and end of each recording. ND96 served as the standard bath solution (composition in millimolar: 96 NaCl, 2 KCl, 1.8 CaCl_2_, 1 MgCl_2_, 5 HEPES; pH 7.4, adjusted with Tris). To assess Na^+^ self-inhibition in Fig. S6, modified bath solutions with either 1 mM Na^+^ (composition in mM: 1 NaCl, 109 mM NMDG-Cl, 2 KCl, 1.8 CaCl_2_, 1 MgCl_2_, 5 HEPES; pH 7.4, adjusted with Tris) or 110 mM Na^+^ (composition in mM: 110 NaCl, 2 KCl, 1.8 CaCl_2_, 1 MgCl_2_, 5 HEPES; pH 7.4, adjusted with Tris) were used, and the flow rate of the bath solution was increased to approximately 10 ml/min to achieve rapid and reproducible solution exchanges at the oocyte, as previously reported (26, 51). Bath solution exchanges with a gravity-fed system were controlled by a magnetic valve system (ALA VM8; ALA Scientific Instruments). Measurements were conducted at room temperature using a continuous holding potential of −60 mV.

### Cell surface biotinylation and Western blot analysis

To separate cell surface proteins from intracellular proteins, a biotinylation approach was used, essentially as described previously (48, 73, 74, 75, 76, 77). Following SDS-PAGE and semidry electroblotting, N-terminal cleavage fragments of α-ENaC, and C-terminal cleavage fragments of β-, γ-, and δ-ENaC were detected using subunit-specific antibodies (Pineda Antibody Service) (28, 48, 68, 69) at a dilution of 1:5000, and horseradish peroxidase-labeled secondary goat anti-rabbit antibodies (Santa Cruz Biotechnology) at a dilution of 1:50,000. Specificity of antibodies was validated with samples from oocytes without injection of cRNA encoding the respective ENaC subunits, as indicated in the corresponding figure legends. To validate the separation of cell surface proteins from intracellular proteins by biotinylation, blots were stripped and reprobed with a polyclonal rabbit anti-β-actin antiserum (Sigma-Aldrich) at a dilution of 1:5000.

### Molecular dynamics simulations

Model systems with simulation box dimensions of approximately 114 × 114 × 114 Å were prepared using 3D periodic boundaries with the Schrödinger Desmond module. Model systems included atoms from αβγENaC or δβγENaC ECLs, were obtained from published cryo-EM structures (PDB-ID: 6WTH, 9BLR; (34, 35)) and prepared by adding missing hydrogens and by capping N- and C-terminal ends with NMA or ACE, respectively. In addition, model systems contained water molecules (simple point charge model), sodium chloride (145 mM), and sodium counterions to achieve electroneutrality. MD simulations were performed, similar as described previously (69), in the NPT ensemble at 1 atm and 310 K using Schrödinger Desmond with the OPLS4 force field. Four simulations (two replicates for αβγENaC and δβγENaC, respectively), each lasting 500 ns with sampling intervals of 100 ps, were conducted. All dynamics simulations started with Desmond's default stepwise relaxation protocol for NPT ensembles over 160 ps. Afterwards, the temperature was maintained at 310 K using the Nosé-Hoover thermostat with a relaxation time of 1 ps. The isotropic pressure of 1.013 bar was sustained with the Martyna-Tobias-Klein barostat, which had a relaxation time of 2 ps. The Coulomb interaction cutoff radius was set to 9 Å. The integration timestep for the reversible reference system propagator algorithm was 2, 2, and 6 fs for bonded, near, and far interactions, respectively. Since the resolved structures include only ENaC ECLs, a positional restraint with a force constant of 100 kcal/mol/Å^2^ was applied to the N- and C-terminal ends of the resolved ECL.

The conformational stability of ENaC during MD simulations was evaluated by calculating Root Mean Square Deviation (RMSD) from the initial structure. To analyze interactions between the P1 or P1/P2-domains and the rest of the ion channel, MD trajectories were examined using the analysis module of the Schrödinger Python Application Programming Interface (API). Structure visualizations were created with UCSF Chimera, developed by the Resource for Biocomputing, Visualization, and Informatics at the University of California, San Francisco, with support from the National Institutes of Health P41-GM103311 (78). The molecular surface calculation was performed using the MSMS package with a probe radius of 1.4 Å and a vertex density of 2 vertices/Å^2^ (79).

### Data analysis and statistical tests

Electrophysiological recordings were analyzed using the software Nest-o-patch (https://sourceforge.net/projects/nestopatch/) developed by Dr Viatcheslav Nesterov (Institute of Cellular and Molecular Physiology, Friedrich-Alexander-Universität Erlangen-Nürnberg, Erlangen, Germany). N indicates the number of different batches of oocytes, and n indicates the number of individual oocytes studied per experimental group. Data are shown as mean ± SD. The normal distribution of data was evaluated using the D'Agostino-Pearson omnibus or Shapiro-Wilk test. Kruskal-Wallis with Dunn’s Test for Multiple Comparisons, or one-way ANOVA with Bonferroni’s *post hoc* test, were employed to determine statistical significance, as specified in figure legends. Statistical analysis and graphical representation of results were performed using the R environment for statistical computation version 4.0.3 (https://www.R-project.org/).

## Data availability

All data are contained within the manuscript and the supporting information.

## Supporting information

This article contains supporting information.

## Conflict of interest

The authors declare that they have no conflicts of interest with the contents of this article.
