# Supplementary material for: Distinct functional roles of the GRIP domains in α-, β-, γ-, and δ-ENaC
Source: J Biol Chem. 2026 Jul 16;302(9):113336. doi: 10.1016/j.jbc.2026.113336 (PMC13488063; doi:10.1016/j.jbc.2026.113336)
Supplement: Supplementary file 1 [file mmc1.pdf]

Distinct functional roles of the GRIP domains in α-, β-, γ- and δ-ENaC

– Supporting Information –

Alexandr V. Ilyaskin, Alicia Kißler, Franziska Schreiber, Christoph Korbmacher, and Florian Sure

Table of Contents

Figure S1 Conformational stability of the P1 segments (in α-, γ-, and δ-ENaC) and P1/P2 segment (in β-ENaC) of the GRIP domains assessed using MD simulations.
Figure S2 Analysis of interactions from the second MD simulation replicas.
Figure S3 Western blot analysis of γ-ENaC constructs with the mutated P1 binding site.
Figure S4 Western blot analysis of γ-ENaC constructs with the mutated P1 segment.
Figure S5 Western blot analysis of α-ENaC constructs with the mutated P1 binding site.
Figure S6 Alanine substitution of aromatic residues within the P1 binding site of α-ENaC (αF226, αW251, αH255, αY447) enhances Na^+ self-inhibition.
Figure S7 Thrombin failed to cleave selected engineered sites in β- and δ-ENaC.
Figure S8 Preventing the release of the inhibitory tract from γ-ENaC abolishes the stimulatory effect of chymotrypsin on αβγ-ENaC.
Figure S9 Original uncropped images of the same western blots as shown in Figure 7.
Figure S10 The intact (wildtype) γ-subunit is crucial for proteolytic ENaC activation in both the δβγ- and αβγ-configurations.

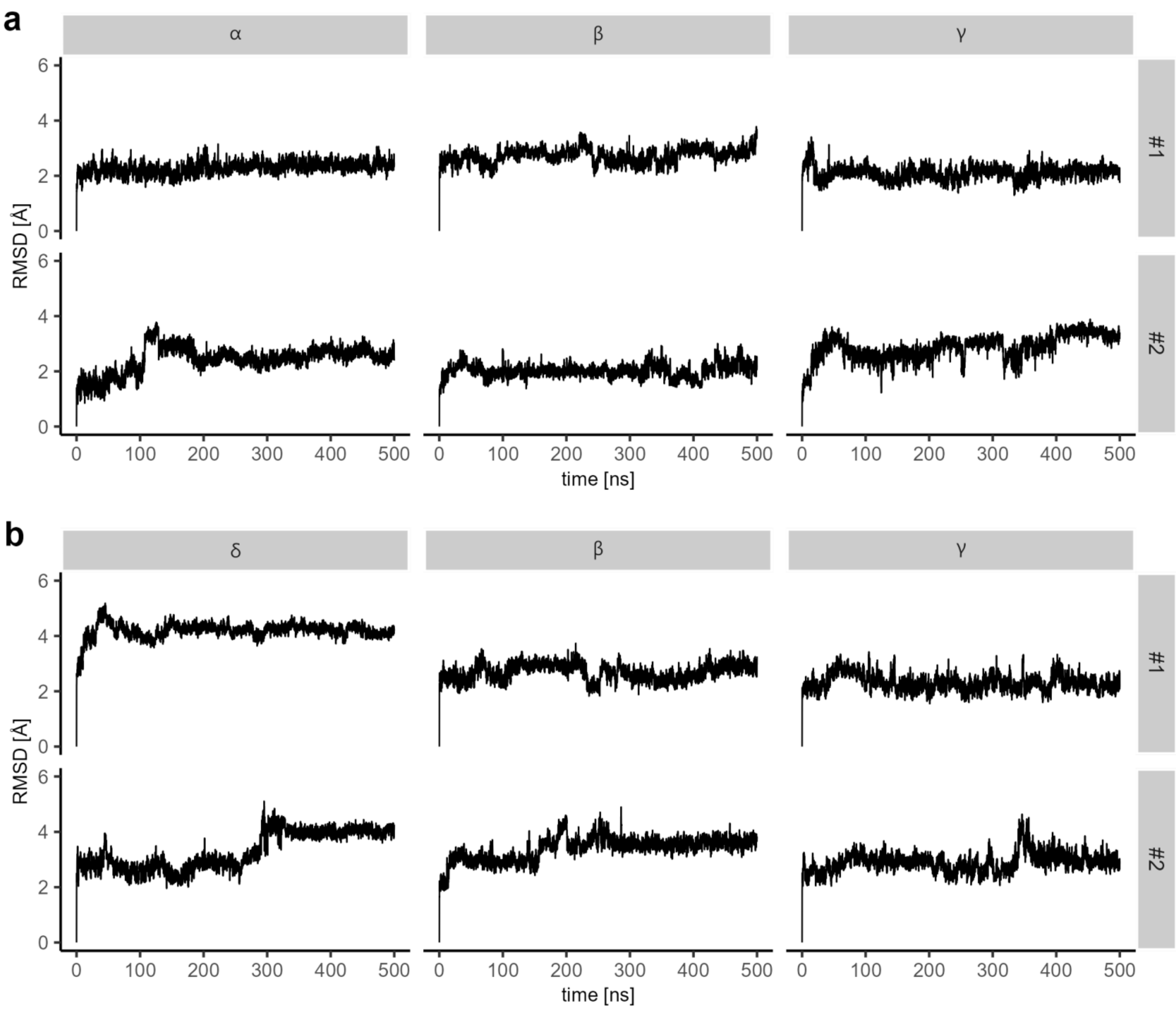

Fig. S1 Conformational stability of the P1 segments (in α-, γ-, and δ-ENaC) and P1/P2 segment (in β-ENaC) of the GRIP domains assessed using MD simulations. Root mean square deviation (RMSD, Å) was calculated for all atoms of the P1 or P1/P2 segments during the first (#1) and second (#2) 500-ns replica MD simulations of αβγ-ENaC (**a**) or δβγ-ENaC (**b**) in each MD frame against the coordinates of the same atoms in the first MD frame. Notably, after a rapid initial shift in RMSD due to equilibration of the structure, the values remained relatively stable, with RMSD fluctuations not exceeding 2 Å.

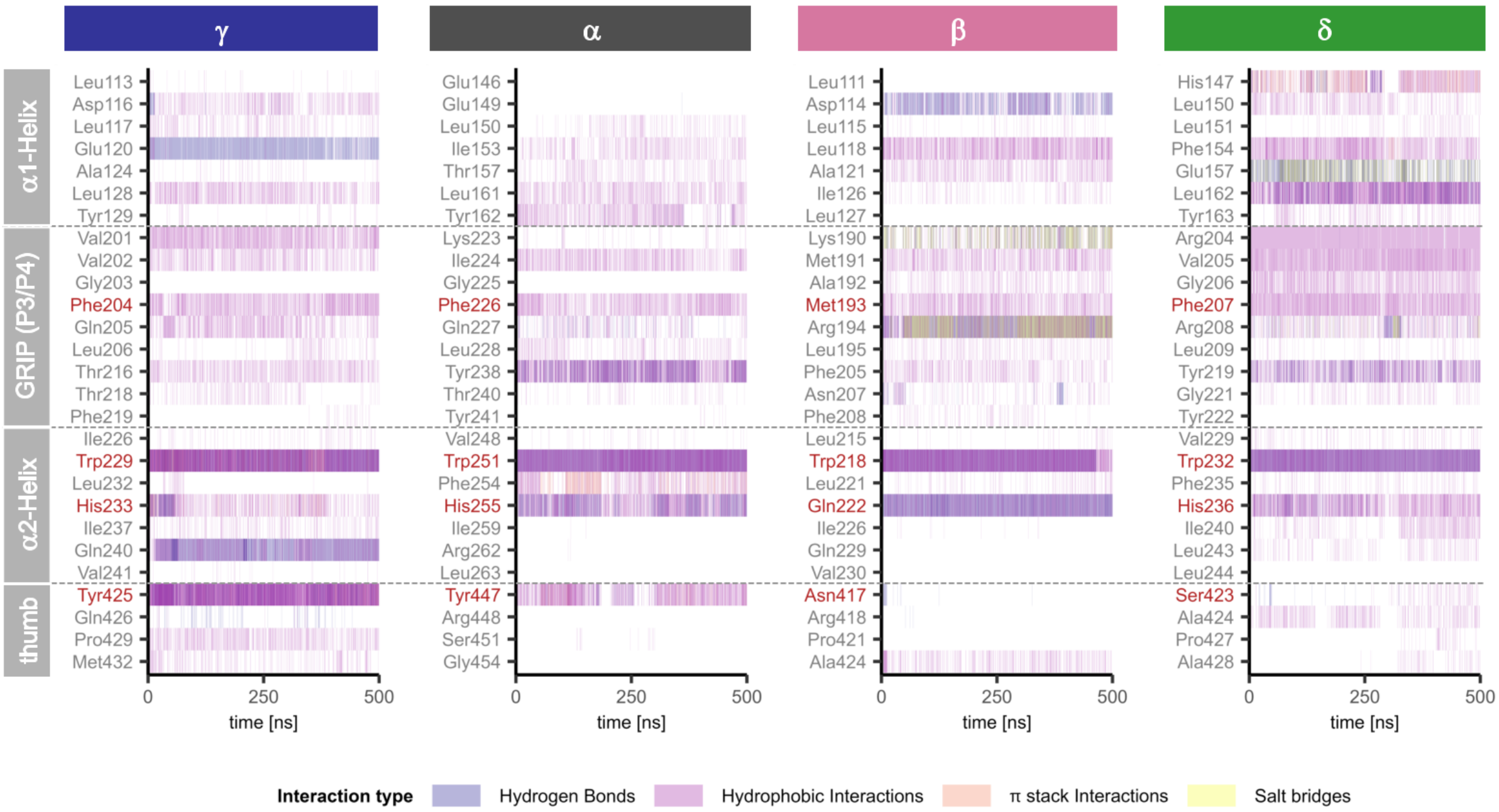

Fig. S2 Analysis of interactions from the second run of MD simulations of αβγ- and δβγ-ENaC. The data are presented in the same manner as described in the figure legend of Fig. 1**b**. The results were similar to those obtained in the corresponding first run of MD simulations shown in Fig. 1**b**.

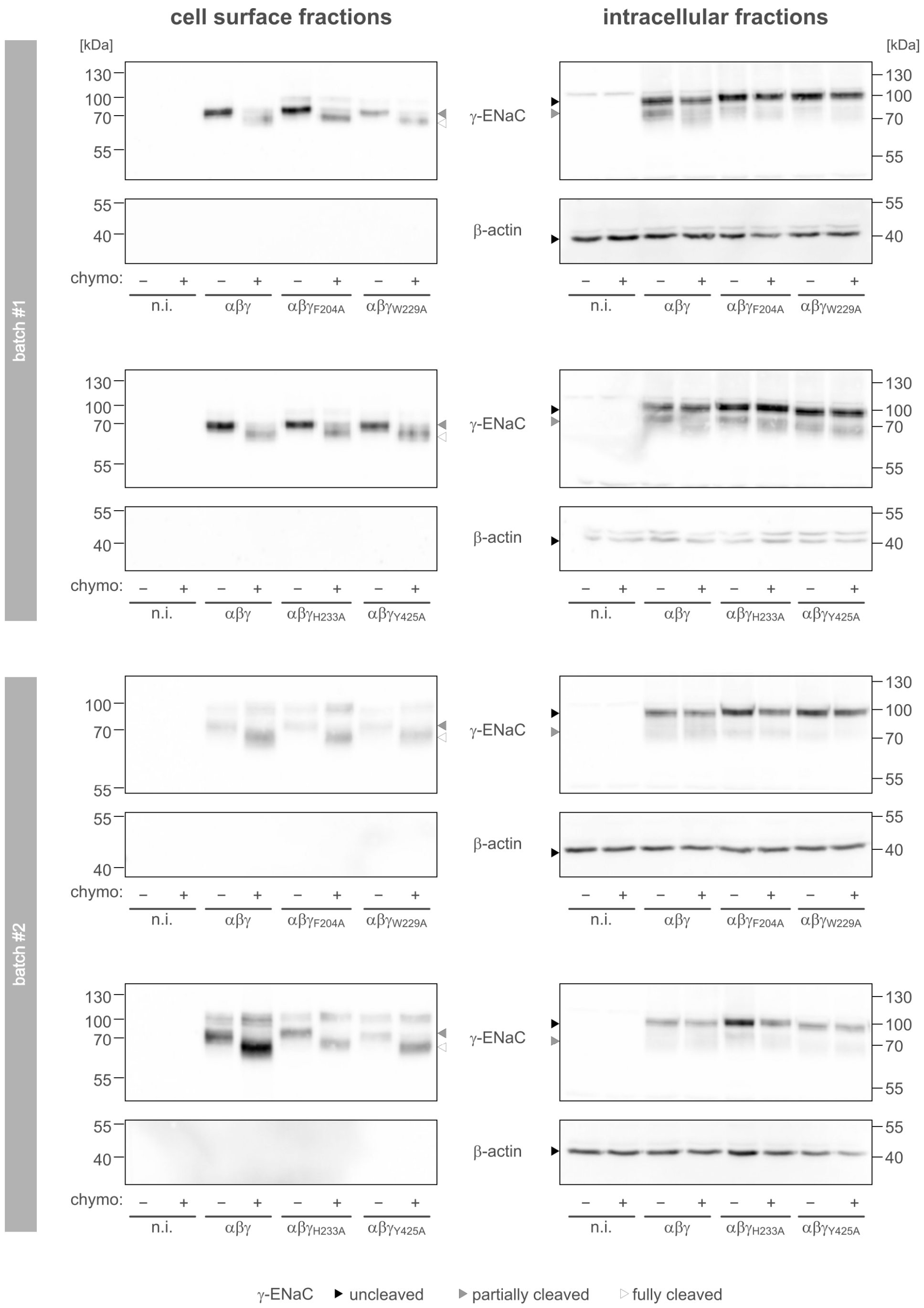

Fig. S3 Western blot analysis of γ-ENaC constructs with the mutated P1 binding site. Cell surface (*left panels*) or intracellular (*right panels*) expression of γ-ENaC and β-actin are shown as indicated. β-actin was detected in the same blots after γ-ENaC detection and stripping, to validate separation of cell surface and intracellular proteins. Oocytes from the first (#1) and second (#2) batch were injected with 0.2 ng/subunit/oocyte cRNA encoding wildtype α- and β-ENaC together with wildtype or mutant γ-ENaC, as specified below the blots. Oocytes were incubated in ND96 without (−) or with (+) 2 µg/ml chymotrypsin for 3 min before biotinylation. γ-ENaC was detected using an antibody against its C-terminal epitope. Non-injected oocytes (n.i.) served as a control for γ-ENaC detection. Uncleaved, partially cleaved, and fully cleaved γ-ENaC are indicated by black-filled, gray, and open arrowheads, respectively. Samples from each oocyte batch were analysed in two separate gels/blots: samples corresponding to different γ-ENaC mutants were analysed once, whereas the n.i. and αβγ-ENaC samples used as control groups were analysed twice (replicated in each gel/blot).

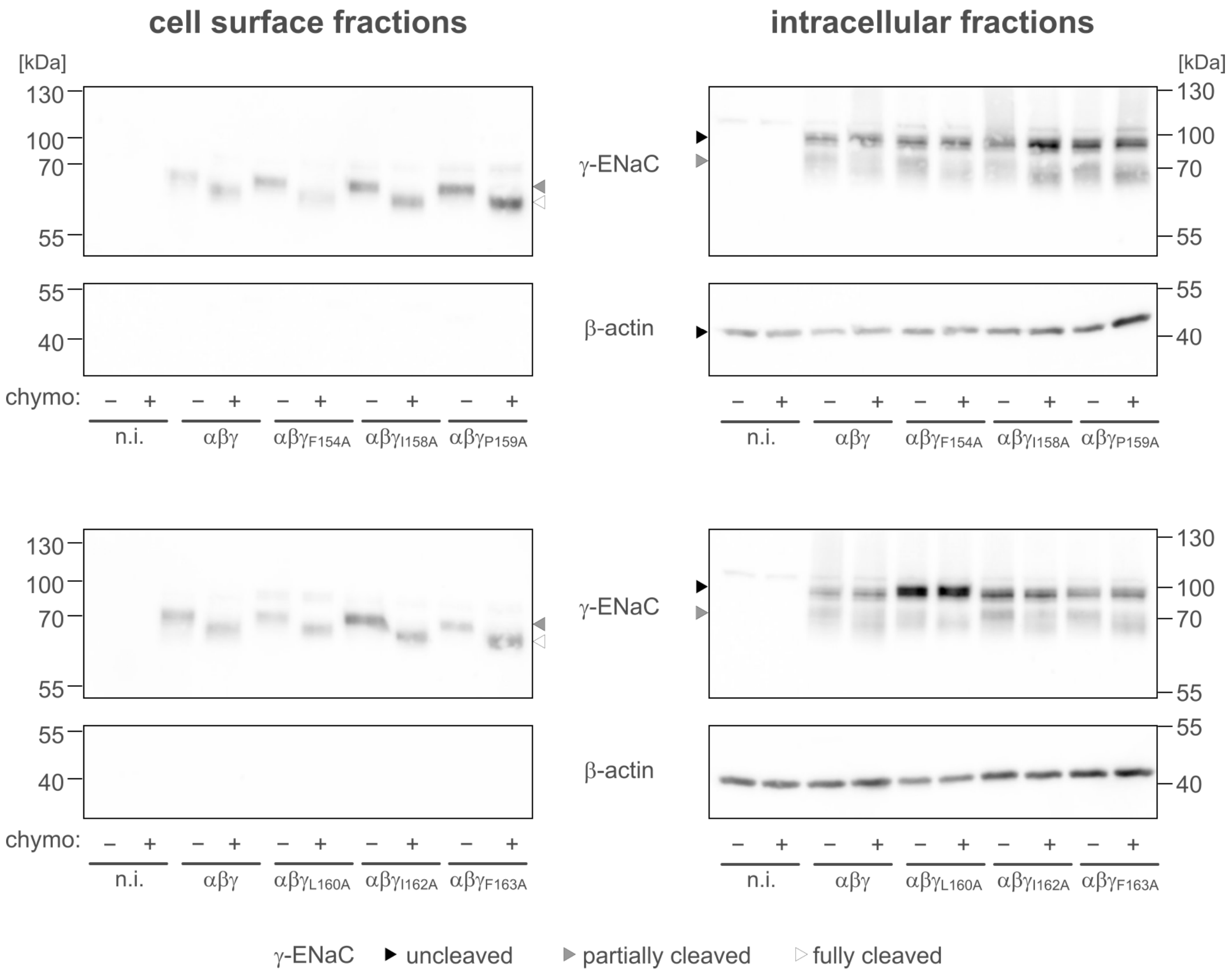

Fig. S4 Western blot analysis of γ-ENaC constructs with the mutated P1 segment. Cell surface (*left panels*) or intracellular (*right panels*) expression of γ-ENaC and β-actin are shown as indicated. β-actin was detected in the same blots after γ-ENaC detection and stripping, to validate separation of cell surface and intracellular proteins. Oocytes from one batch were injected with 0.2 ng/subunit/oocyte cRNA encoding wildtype α- and β-ENaC together with wildtype or mutant γ-ENaC, as specified below the blots. Oocytes were incubated in ND96 without (−) or with (+) 2 µg/ml chymotrypsin for 3 min before biotinylation. γ-ENaC was detected using an antibody against its C-terminal epitope. Non-injected oocytes (n.i.) served as a control for γ-ENaC detection. Uncleaved, partially cleaved, and fully cleaved γ-ENaC are indicated by black-filled, gray, and open arrowheads, respectively. Samples were analysed in two separate gels/blots: samples corresponding to different γ-ENaC mutants were analysed once, whereas the n.i. and αβγ-ENaC samples used as control groups were analysed twice (replicated in each gel/blot).

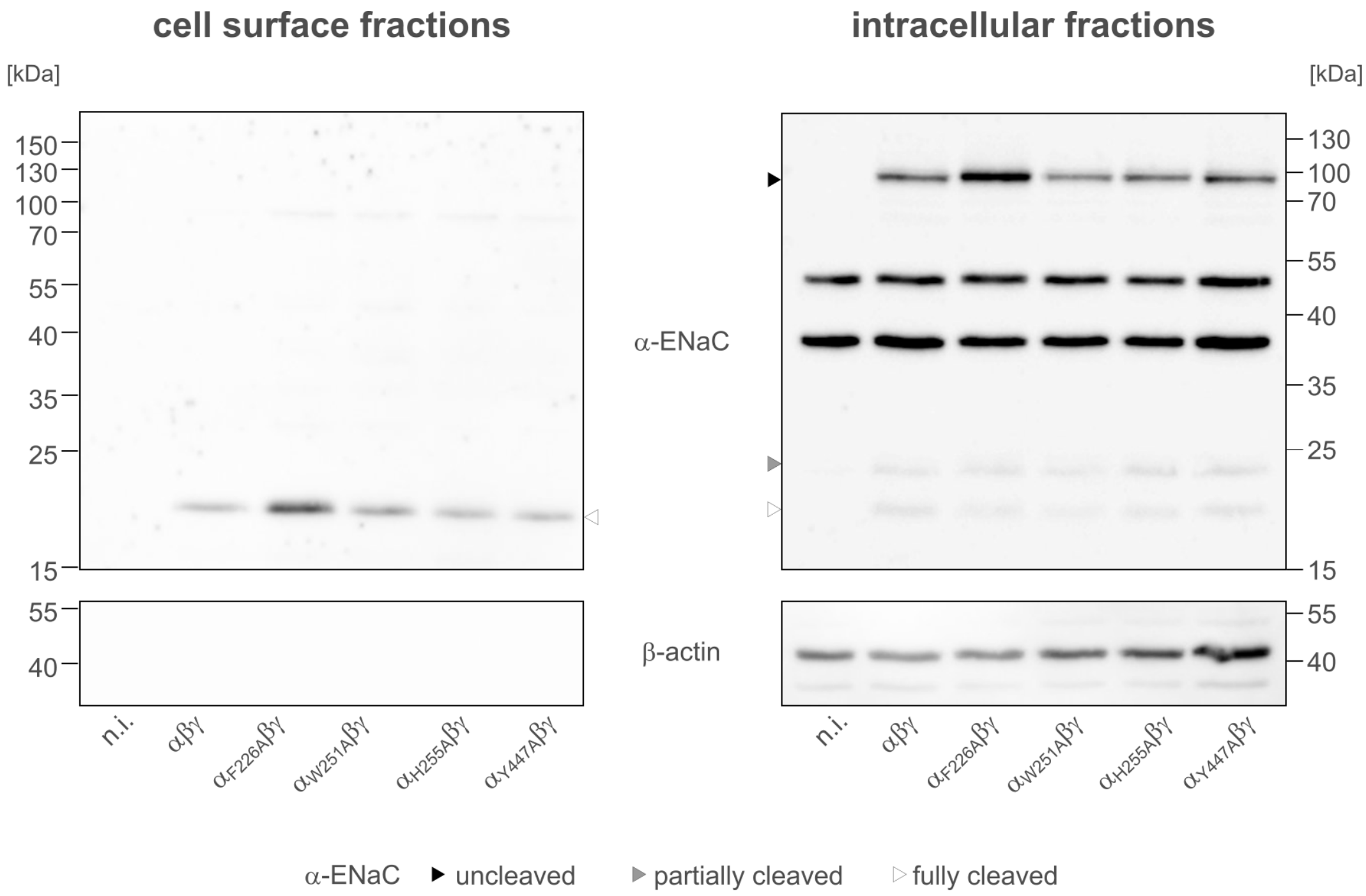

Fig. S5 Western blot analysis of α-ENaC constructs with mutated P1 binding site. Cell surface (*left panels*) or intracellular (*right panels*) expression of α-ENaC and β-actin are shown as indicated. β-actin was detected in the same blots after α-ENaC detection and stripping, to validate separation of cell surface and intracellular proteins. Oocytes from one batch were injected with 0.2 ng/subunit/oocyte cRNA encoding wildtype β- and γ-ENaC together with wildtype or mutant α-ENaC, as specified below the blots. α-ENaC was detected using an antibody against its N-terminal epitope. Non-injected oocytes (n.i.) served as control for α-ENaC detection. Uncleaved, partially cleaved, and fully cleaved α-ENaC are indicated by black-filled, gray, and open arrowheads, respectively.

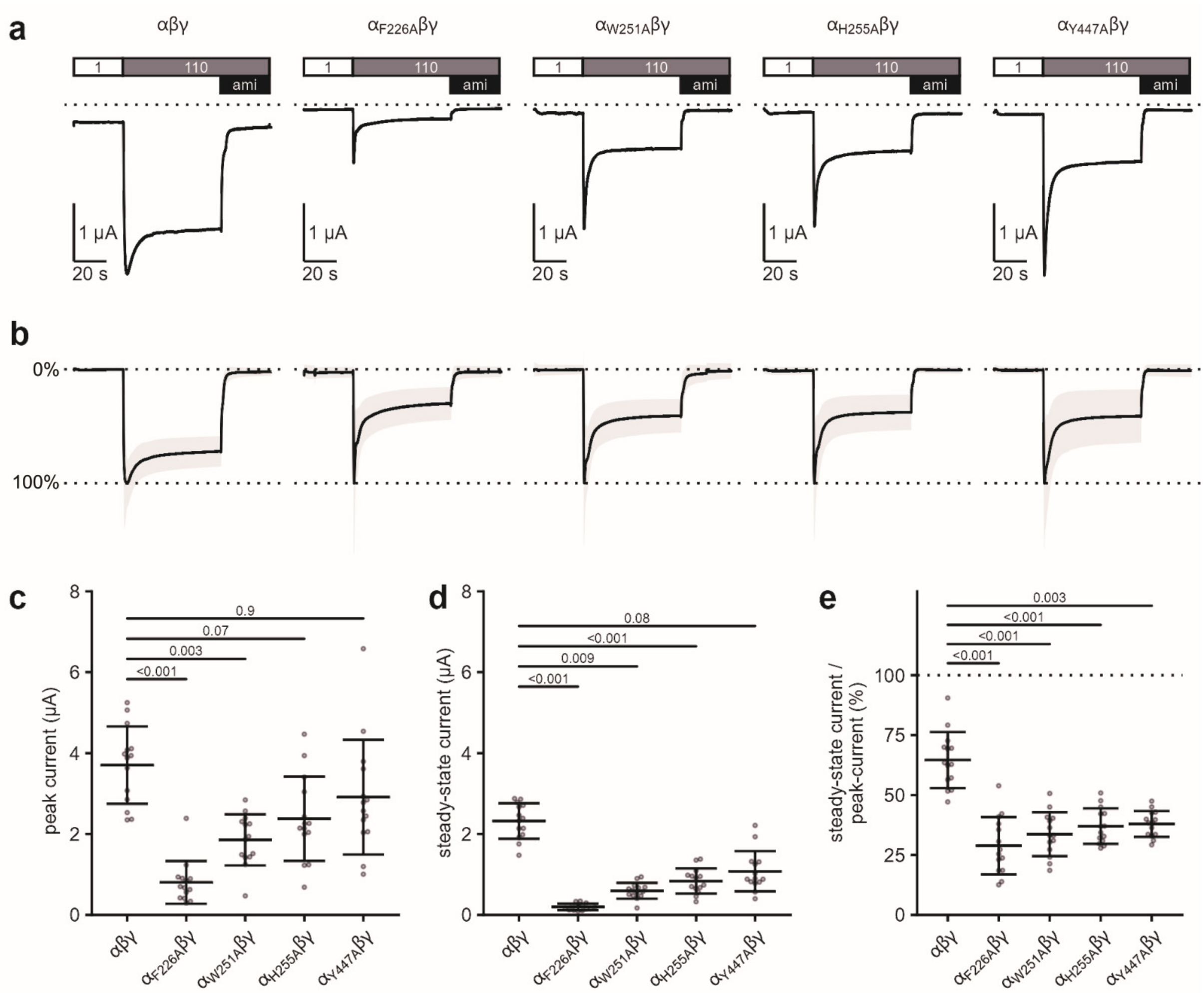

Fig. S6 Alanine substitution of aromatic residues within the P1 binding site of α-ENaC (αF226, αW251, αH255, αY447) enhances Na^+ self-inhibition. **a** Representative whole-cell current traces for oocytes co-expressing human wildtype β- and γ-ENaC together with the wildtype α-subunit (αβγ) or the corresponding α-ENaC mutant ($\alpha_{F226A}\beta\gamma$, $\alpha_{W251A}\beta\gamma$, $\alpha_{H255A}\beta\gamma$, or $\alpha_{Y447A}\beta\gamma$) are shown. The bath solution contained either 1 mM or 110 mM Na^+, as indicated by white and grey bars, respectively. In addition, amiloride (ami, 2 µM) was present in the bath solution as indicated by black bars. Dotted lines correspond to the zero current level. **b** Normalized average current traces from similar experiments as shown in **a**. Before averaging, individual traces within each experimental group were normalized to the mean current measured in the presence of amiloride (0%) and to the mean peak current reached upon switching from 1 mM to 110 mM NaCl (100%) in oocytes from the same group. Mean values are shown by a black line, the surrounding grey area represents SD (N = 1, n = 14; N indicates the number of different batches of oocytes, n indicates the number of individual oocytes studied per experimental group). **c, d** Summary of peak (**c**) or steady-state (**d**) ΔI_{ami} values. Mean ± SD and datapoints for individual oocytes are shown. **e** To assess Na^+ self-inhibition, the steady-state current was divided by the peak current for each individual oocyte. Mean ± SD and datapoints for individual oocytes are shown. *p* values in **c-e** were calculated using Kruskal-Wallis with Dunn's post-hoc test.

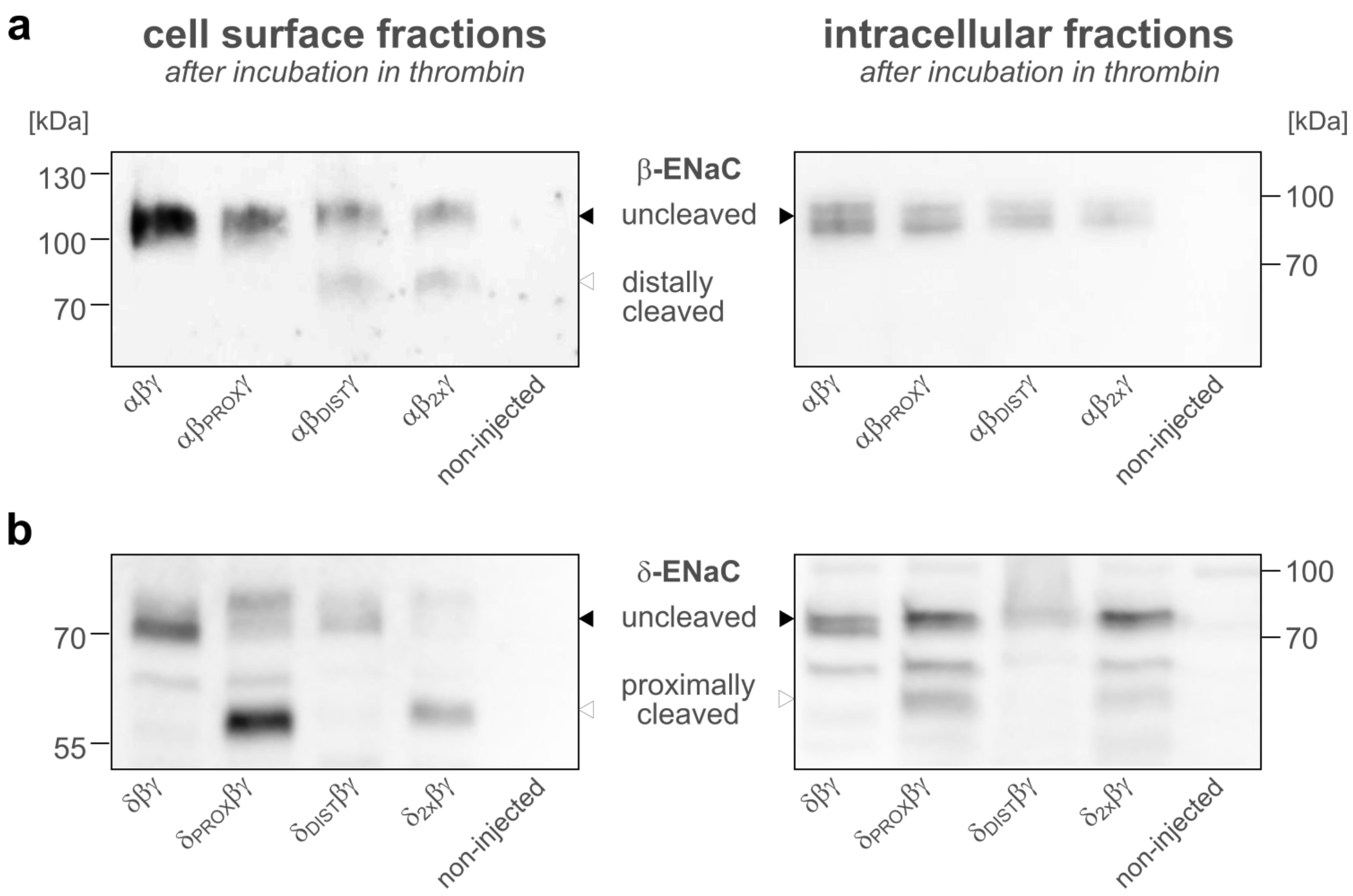

Fig. S7 Thrombin failed to cleave selected engineered sites in β- and δ-ENaC. a Western blot showing cell surface (*left panel*) or intracellular (*right panel*) expression of β-ENaC in oocytes from one batch co-expressing wildtype α- and γ-ENaC together with one of the indicated β-ENaC variants: wild-type (β), with a thrombin cleavage site inserted proximal (β_{PROX}), distal (β_{DIST}), or both proximal and distal (β_{2X}) to the P1/P2 segment. Oocytes were incubated with 200 nM thrombin in ND96 for 15 min before biotinylation. Cleavage fragments of β-ENaC were detected as described in Fig. 5**a**, **b**. Whereas uncleaved (~110 kDa; black-filled arrowhead) and distally cleaved (~70 kDa; open arrowhead) β-ENaC were detected, the signal corresponding to proximally cleaved β-ENaC was absent. **b** Similar western blot experiments as described in **a**, but showing expression of δ-ENaC in oocytes from one batch co-expressing wildtype β- and γ-ENaC together with one of the indicated δ-ENaC variants: wild-type (δ), with a thrombin cleavage site inserted proximal (δ_{PROX}), distal (δ_{DIST}), or both proximal and distal (δ_{2X}) to the P1 segment. Oocytes were incubated with 200 nM thrombin in ND96 for 15 min prior to biotinylation. Cleavage fragments of δ-ENaC were detected as described in Fig. 8**a**. The blot in the right panel was mirrored vertically after detection to align all groups with the order shown in the left panel. Notably, the proximally cleaved δ-ENaC fragment was already detectable in the intracellular fraction of oocytes expressing δ_{PROX} or δ_{2X}, suggesting cleavage by intracellular proteases rather than by thrombin. No signal corresponding to distally cleaved δ-ENaC was observed.

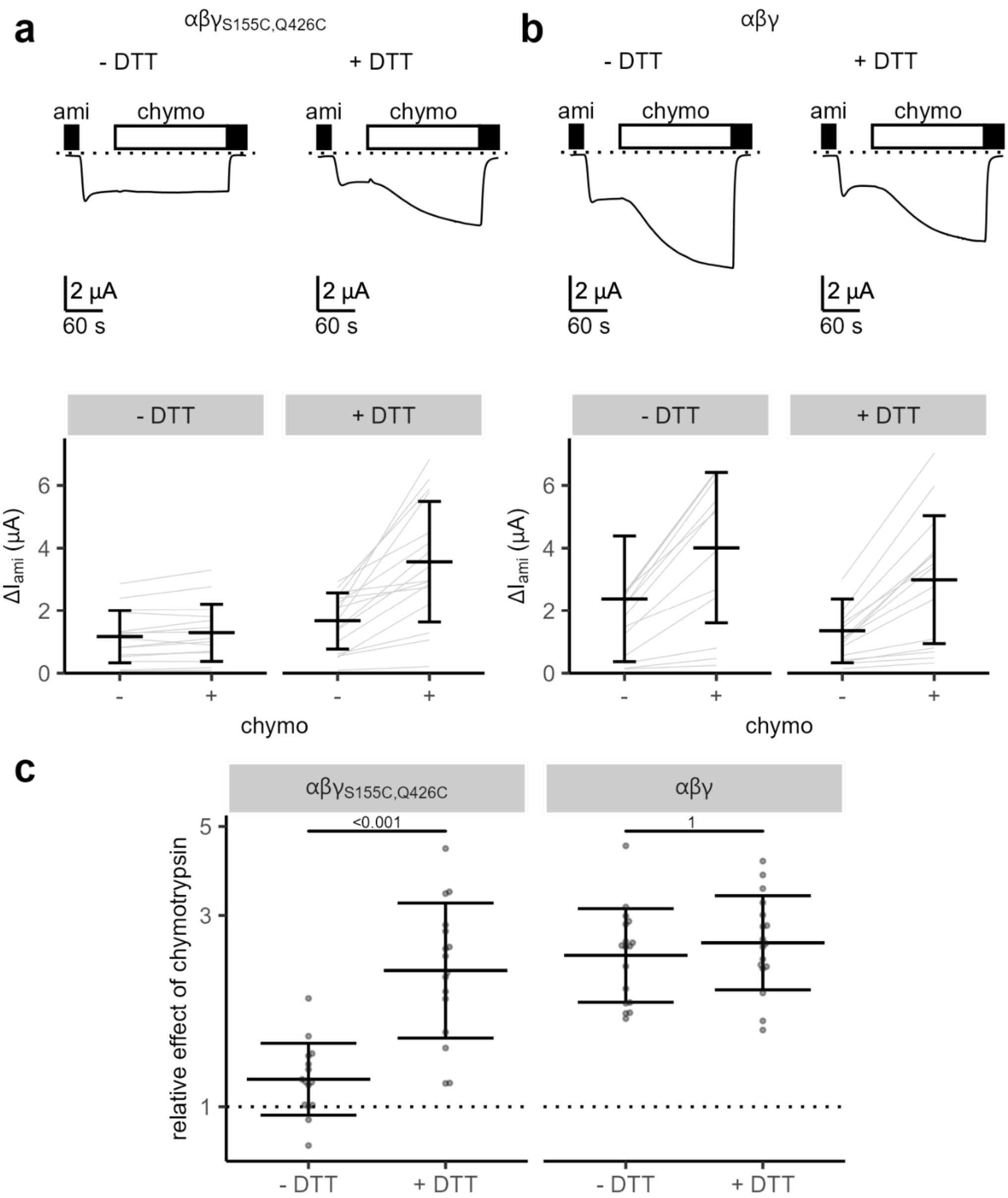

Fig. S8 Preventing the release of the inhibitory tract from γ-ENaC abolishes the stimulatory effect of chymotrypsin on αβγ-ENaC. a, **b** *Upper panels*, A representative whole-cell current trace is shown for an oocyte co-expressing human wildtype α- and β-ENaC together with either the disulfide-locked γ-ENaC mutant ($\alpha\beta\gamma_{S155C,Q426C}$, **a**) or the wildtype γ-subunit (αβγ; **b**). Oocytes were incubated either in ND9 without DTT (- DTT, *left panels*) or with 30 mM DTT (+ DTT, *right panels*) for 15 min before current measurement. Amiloride (ami, 2 µM) and chymotrypsin (2 µg/ml) were present in the bath solution as indicated by black and white bars, respectively. Dotted lines correspond to the zero current level. Lower panels, ΔI_{ami} values were determined before (−) and after (+) chymotrypsin application from similar experiments as shown in the upper panels as described in Figure 2. Gray lines connect data points obtained in an individual oocyte. Mean ± SD are shown in black (**a-b**: N = 3, $15 \leq n \leq 17$; N indicates the number of different batches of oocytes, n indicates the number of individual oocytes studied per experimental group). **c** Relative stimulatory effect of chymotrypsin was determined using ΔI_{ami} values from the same experiments as shown in **a**, **b** and are displayed on a logarithmic scale. Dotted line indicates relative effect of one (no effect). p values were calculated using one-way ANOVA with Bonferroni post-hoc test of log-transformed values.

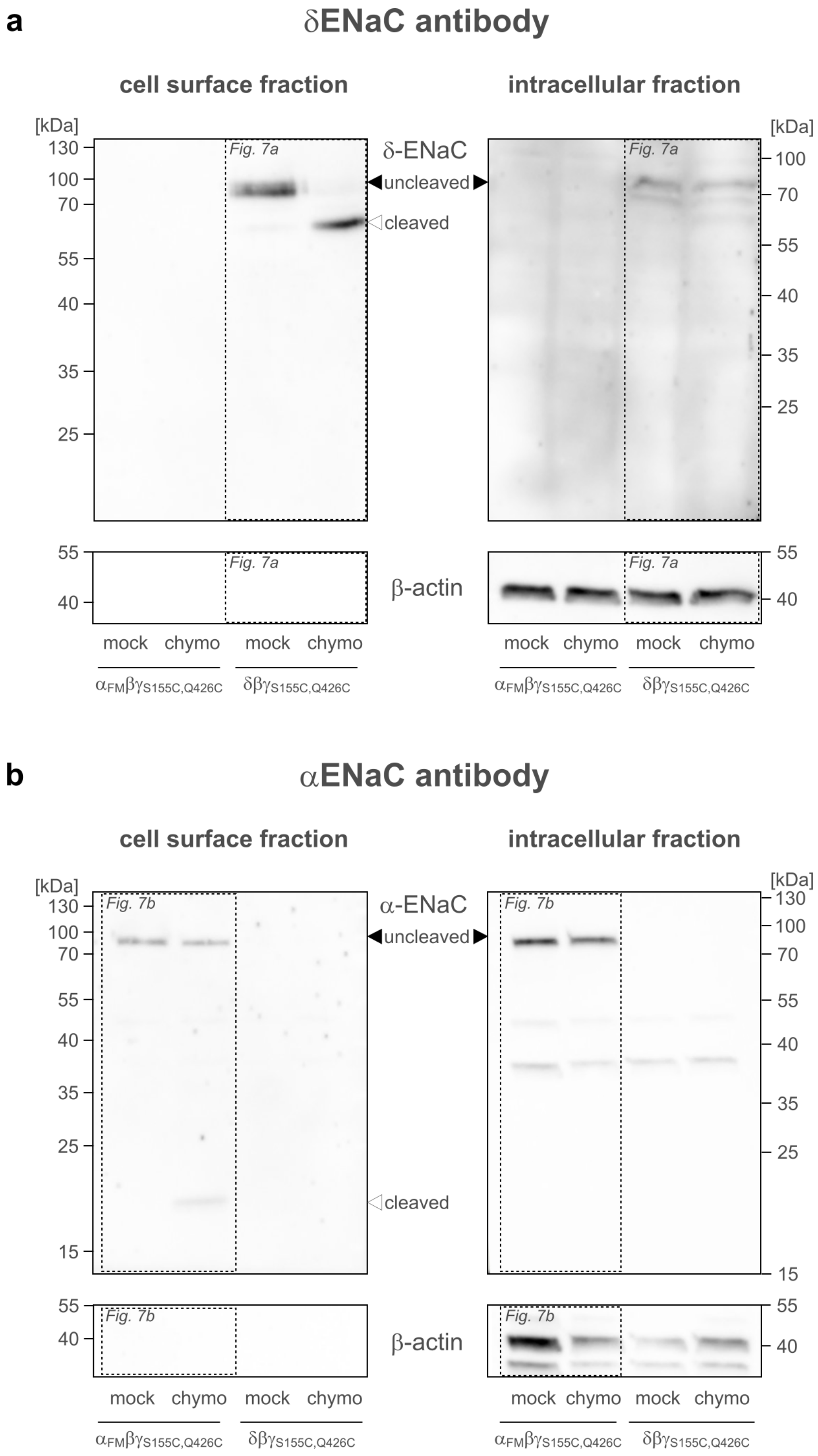

Fig. S9 Original uncropped images of the same western blots as shown in Figure 7. Cell surface (*left panels*) or intracellular (*right panels*) expression of δ-ENaC (**a**) or α-ENaC (**b**) and β-actin are shown as indicated. β-actin was detected in the same blots after ENaC detection and stripping, to validate separation of cell surface and intracellular proteins. δ-ENaC and α-ENaC were detected using antibodies against the C-terminal and N-terminal epitopes, respectively. The specific δ- and α-ENaC signals were detected only in oocytes expressing the corresponding subunit and were absent in oocytes lacking their expression. Parts of the blots shown in Fig. 7**a** and 7**b** are framed by dotted rectangles.

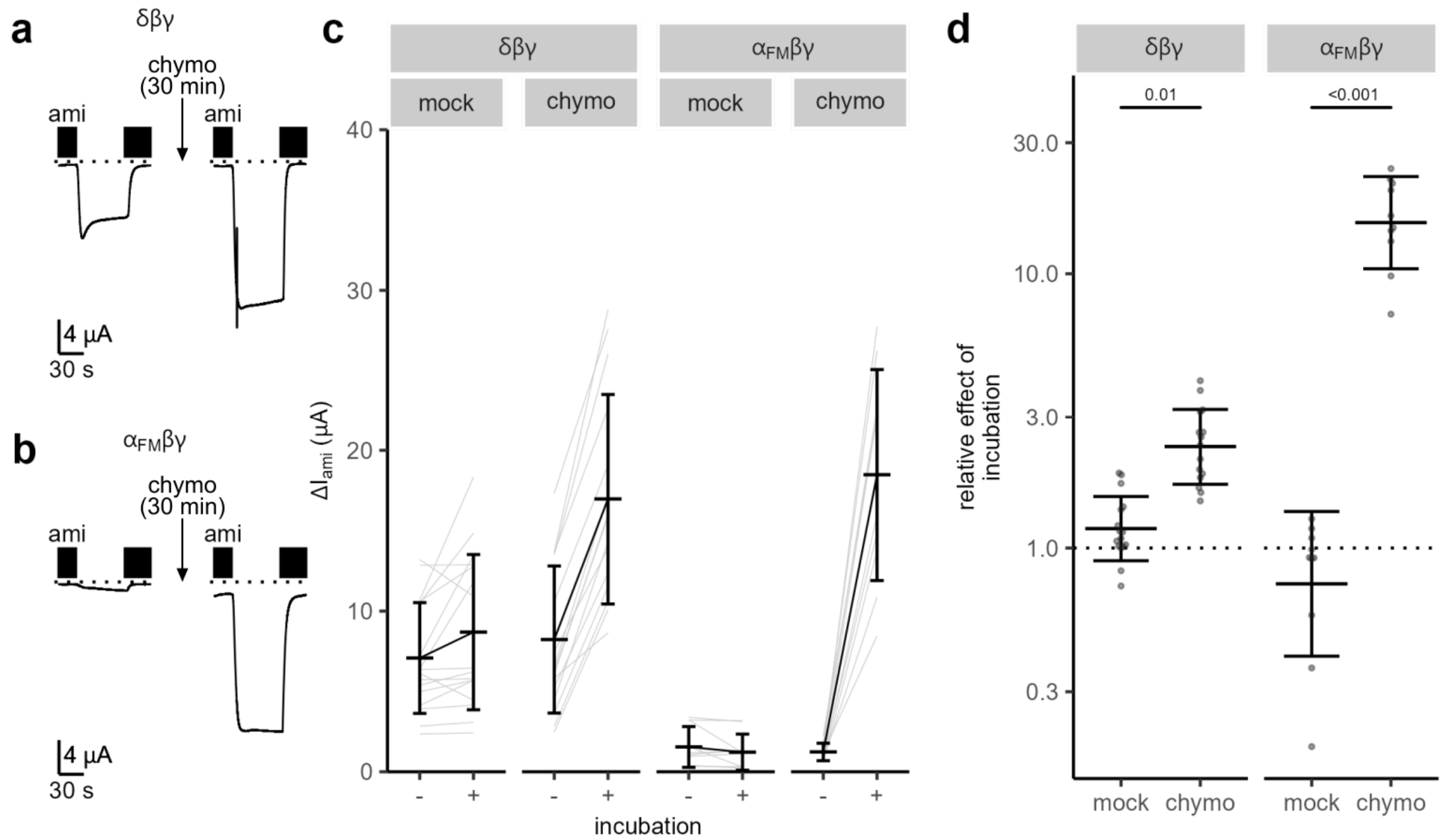

Fig. S10 The intact (wildtype) γ-subunit is crucial for proteolytic ENaC activation in both the δβγ- and αβγ-configurations. a, **b** A representative whole-cell current trace is shown for an oocyte expressing δβγ- (**a**) or α_{FM}βγ-ENaC (**b**) before (*left traces*) and after 30 min of chymotrypsin treatment (*right traces*). α_{FM} corresponds to the α-ENaC mutant in which both proximal ($_{175}$RSRR$_{178}$) and distal ($_{201}$RRAR$_{204}$) furin cleavage sites were mutated to alanines. Amiloride (ami; 100 µM in **a** or 2 µM in **b**) was present in the bath solution as indicated by black bars. Dotted lines correspond to the zero current level. **c** ΔI_{ami} values were determined as described in Figure 2 in chymotrypsin-treated oocytes as shown in **a**, **b** and additionally in mock-treated oocytes before (−) and after (+) correspondent incubation. Experiments were performed in parallel under the same conditions as those shown in Figure 7. Gray lines connect data points obtained in an individual oocyte. Mean ± SD are shown in black ($N = 2$, $10 \leq n \leq 16$; N indicates the number of different batches of oocytes, n indicates the number of individual oocytes studied per experimental group). **d** Relative effect of mock or chymotrypsin incubation on ΔI_{ami} obtained from the same experiments as shown in **c** and displayed on a logarithmic scale. Dotted line indicates relative effect of one (no effect). *p* values were calculated using Kruskal-Wallis with Dunn's post-hoc test of log-transformed values.
